# Discovery of 5-methyl-*N*-(2-arylquinazolin-7-yl)isoxazole-4-carboxamide analogues as highly selective FLT3 inhibitors

**DOI:** 10.1080/14756366.2020.1758689

**Published:** 2020-04-27

**Authors:** Daseul Im, Hyungwoo Moon, Jinwoong Kim, Youri Oh, Miyoung Jang, Jung-Mi Hah

**Affiliations:** College of Pharmacy and Institute of Pharmaceutical Science and Technology, Hanyang University, Ansan, Gyeonggi-do, Korea

**Keywords:** FLT3, FLT3-ITD, FLT3-TKD, quinazoline, selectivity

## Abstract

A series of 4-arylamido 5-methylisoxazole derivatives with quinazoline core was designed and synthesised based on conformational rigidification of a previous type II FMS inhibitor. Most of quinazoline analogues displayed activity against FLT3 and FLT3-ITD. Compound **7d**, 5-methyl-*N*-(2-(3-(4-methylpiperazin-1-yl)-5-(trifluoromethyl)phenyl)quinazolin-7-yl)isoxazole-4-carboxamide, exhibited the most potent inhibitory activity against FLT3 (IC_50_= 106 nM) with excellent selectivity profiles over 36 other protein kinases including cKit and FMS kinase. Compound **7d** was also active in FLT-ITD, with an IC_50_ value of 301 nM, and other FLT3 mutants showing potential as an AML therapeutics.

## 1. Introduction

FLT3, a type of trans-membrane receptor tyrosine kinase expressed on lympho-hematopoietic cells, is an attractive target in acute myeloid leukaemia (AML)^1^. When FLT3 ligand binds to FLT3 kinase, it is activated by auto-phosphorylation, which then activates its multiple downstream signalling pathways, including signal transducer and activator of transcription 5 (STAT5), ras/mitogen-activated protein kinase, and phosphatidylinositol 3-kinase/Akt pathways, playing a key role in cell proliferation, survival, and immune response^2–5^.

**Figure 1.**
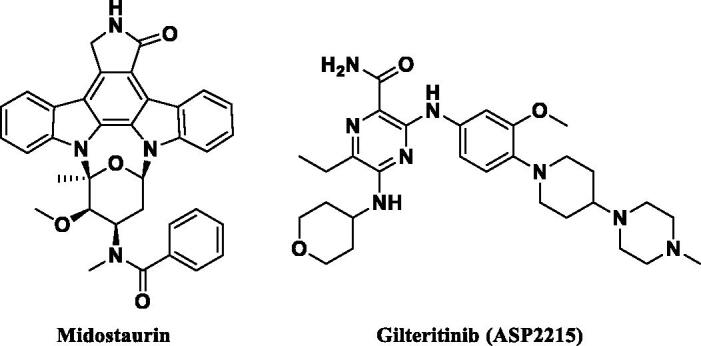
FLT3 inhibitors approved by the FDA as AML treatment.

However, mutation of FLT3 causes its ligand-independent activation and can be classified by location and type, including internal tandem duplication (ITD) and point mutation in the tyrosine kinase domain (TKD). FLT3-ITD mutations are present in 20–30% of AML patients and are especially related to leucocytosis and poor prognosis and point mutation in TKD is found in 5% of AML patients^6^. Despite the significance of seeking AML therapeutics, only a few molecules have been approved, such as midostaurin and gilteritinib (ASP2215), which are depicted in Figure 1^7,8^.

**Figure 2.**
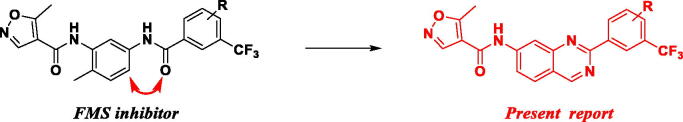
Design of quinazoline derivatives as bioisosteres of the middle phenyl ring-amide-bond.

Protein kinase inhibitors can be classified as type I, II, III, IV and V^9^ based on their binding mode. Among these inhibitors, type II kinase inhibitor can take advantage of selectivity acquisition and exhibits promising potency due to additional interaction with the DFG pocket adjacent to the ATP-binding pocket on top of the hinge hydrogen bond in the ATP site^10^. Previously, we reported very potent type II FMS inhibitors^11^ and selective benzimidazole-incorporated FLT3 inhibitors^12^. Here, we demonstrate that the amide or urea between the middle phenyl ring and the secondary hydrophobic aromatic ring can be rigidified further, introducing a *quinazoline* structure as a bioisostere (Figure 2). The *quinazoline* structures are well-known privileged structures in medicinal chemistry, exhibiting diverse biological activities^13^. However, this is the first attempt to incorporate one in type II PKI modification. Introducing *quinazoline* into type II FMS kinase inhibitor, we could discover a novel FLT3 inhibitor with excellent selective profile.

## 2. Results and discussion

**Scheme 1.**
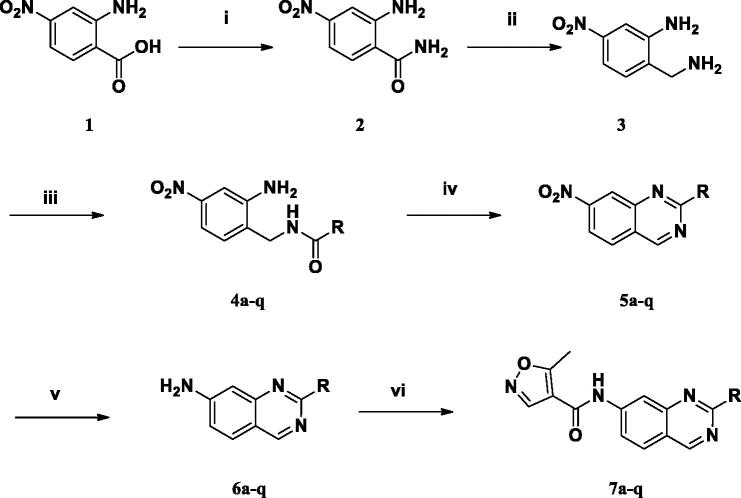
Syntheses of 1*H*-quinazolyl isoxazole-4-carboxamide derivatives. (i) EDC, HOBt, TEA, NH_3_ in MeOH, rt; (ii) BH_3_-THF, reflux; (iii) benzoyl chloride, CH_2_Cl_2_, 0 ^o^C→ rt; (iv) (1) HCl/H_2_O/AcOH, μW, 150 ^o^C, 10 min; (2) *p*-chloranil, toluene, reflux; (v) Fe, AcOH/H_2_O/EtOH, 60 ^o^C; (vi) 5-methylisoxazole-4-carbonyl chloride, TEA, THF, rt.

Scheme 1 shows the general synthetic route for quinazolinyl-isoxazole-4-carboxamide analogues (**7a**–**q**). First, 2-amino-4-nitro-benzoic acid (**1**) was converted to 2-amino-4-nitrobenzamide (**2**) and then reduced to 2-(aminomethyl)-5-nitroaniline (**3**) using borane-tetrahydrofuran. Then, the benzyl amine was coupled with various benzoyl chlorides to produce carboxamide derivatives (**4a**–**q**); subsequently, benzamide was treated with concentrated HCl in acetic and subjected to microwave irradiation at 150 °C for cyclisation to yield a dihydroquinazoline compound. Without further purification, they were treated with *p*-chloranil oxidising agent to obtain quinazoline derivatives (**5a**–**q**) as core intermediates. Next, the nitro group was reduced to amine (**6a**–**q**) using Fe catalyst and was then coupled with isoxazole chloride to produce the final quinazolinyl-isoxazole-4-carboxamides (**7a**–**q**).

All quinazoline compounds, **7a**–**q**, were evaluated for their activity against FLT3 kinase and FLT3-ITD mutation and the results are shown in Table 1. Most of the synthesised compounds exhibited selective activity against FLT3, particularly those incorporating the piperazine moiety. Among the compounds evaluated, **7d** showed the most potent activity against FLT3 with an IC_50_ value of 106 nM, and FLT3-ITD with an IC_50_ value of 301 nM. Structure activity relationships (SARs) were inferred from the data.

**Table 1.** Enzymatic activity of 5-methyl-*N*-(2-arylquinazolin-7-yl) isoxazole-4-carboxamide analogues.

								

In our previous work, benzimidazole compounds retained their activity against FLT3 regardless of presence of 1,3,5-substituted or 1,3,4-substituted benzoic acid, and the activity was determined as piperazine > imidazole > morpholine substituents^12^. We optimised quinazoline derivatives based on the observation of previous benzimidazole derivatives. Those with methyl piperazine or morpholine as the phenyl substitution group (**7d** and **7b**) were more potent about 2- to 5-fold (IC_50_ values of 0.106 and 3.98 µM, respectively) compared to corresponding benzimidazole series (IC_50_ values of 0.495 and 7.94 µM), and **7c** displayed better potency (IC_50_ value of 1.58 µM) than that of benzimidazole (IC_50_ value of 2.33 µM). Introduction of 3,5-disubstituted benzoic acid through quinazoline connection maintained the activity (**7b**, **7c**, **7d, 7e, 7n),** but quinazoline compound with 1,3,4-substituted benzoic acid (**7a**) and one with pyrazole (**7h**) caused loss of activity towards FLT3.

**Figure 3.**
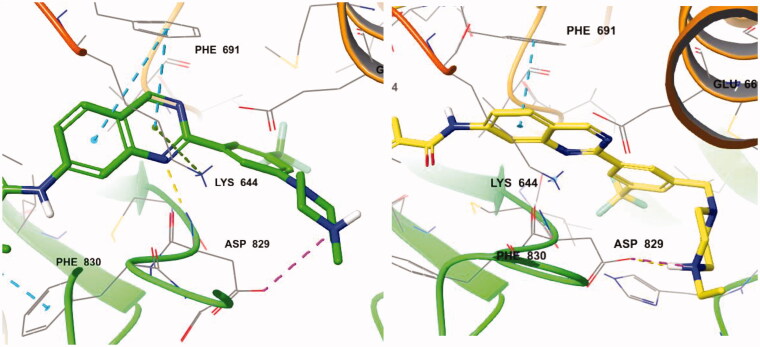
(Left) Compound **7d** (green) at the active site of FLT3 (PDB: 4RT7); (right) **7e** (yellow) at the active site of FLT3 (PDB: 4RT7).

**Figure 4.**
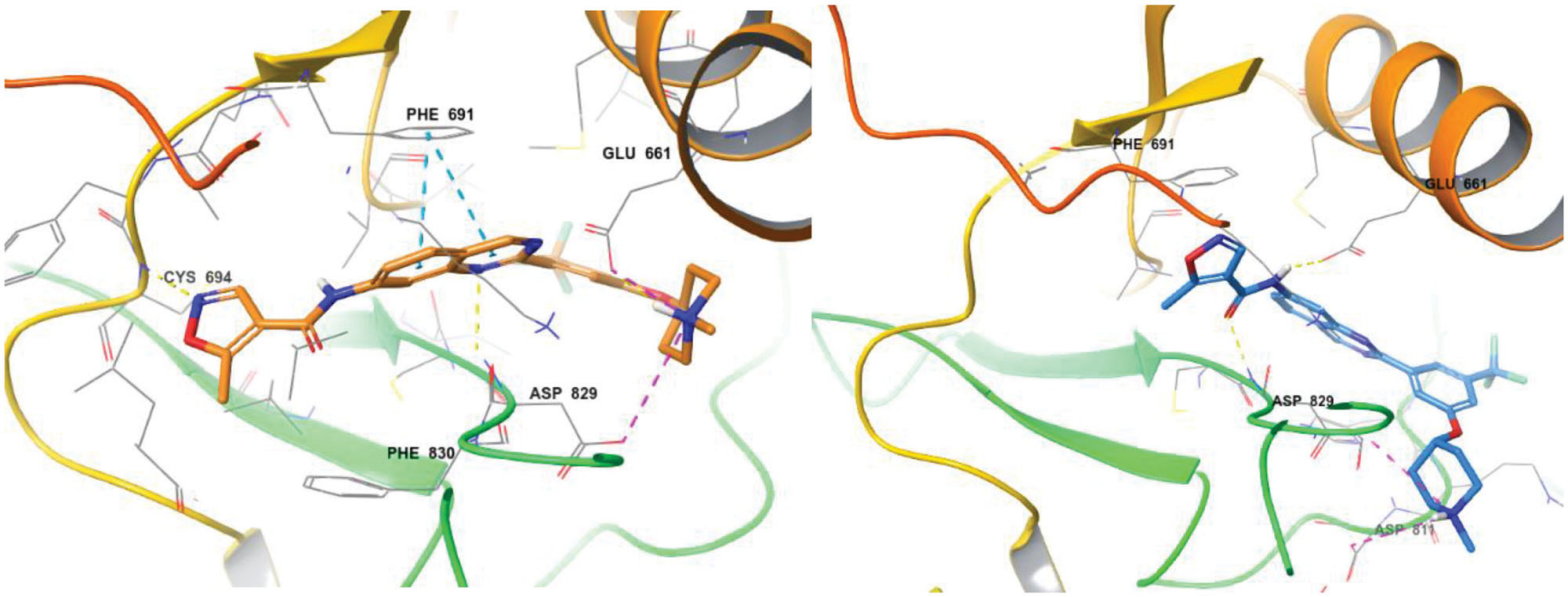
(Left) Compound **7n** with equatorial O linkage (orange) at the active site of FLT3 (PDB: 4RT7); (right) compound **7n** with axial O linkage (azure) at the active site of FLT3 (PDB: 4RT7).

With the result of **7d**, we synthesised compound **7e** to optimise the linkage between the phenyl group and the piperazine moiety. Although inhibitory activity towards FLT3 was retained, **7e** exhibited decreased activity, about 10-fold less than that of **7d**. On the predicted binding mode of **7d**, strong ionic interaction between the protonated nitrogen of the piperazine and Asp829 might enhance its binding affinity. Almost the same ionic interaction seems possible in case **7e**, but the ionic interaction might push the whole compound slightly out of the active site due to its length, resulting in loss of multiple interactions such as hydrogen bonding with Asp829, π–π interaction with Phe691, and π–cation interaction with Lys644 (Figure 3). We also replaced piperazine with a piperidine moiety (**7n**) to investigate the role of nitrogen in the piperazine structure. The IC_50_ value of **7n** was 3.59 µM, similar but weaker than **7e** despite their similar structures. Our docking study showed that one conformer of **7n** with equatorial O linkage bound with FLT3 similarly to compound **7e**, but the other conformer with axial O linkage was not suitable to bind tightly to the active site because of its non-linear piperidine moiety (Figure 4).

We diversified our quinazoline compounds for further optimisation, introducing a halogen group (**7f**, **7g**, and **7i**), *tert*-butyl isoxazole (**7l**), and styrenyl group (**7j** and **7k**). However, only **7j** exhibited competitive activity against FLT3, with an IC_50_ value of 4.7 µM. In addition, we tried to introduce isoxazole, indazole, acetyl piperidine, and pyridine (**7l**–**q**). Compound **7m** showed activity against FLT3, with an IC_50_ value of 0.79 µM. Compounds **7l**, **7o**, **7p,** and **7q** did not show inhibitory activity towards FLT3 or were very weak. The *tert*-butyl isoxazole, acetyl piperidine, and pyridine moieties were less basic than other moieties (piperazine, morpholine, imidazole, indazole, and piperidine). For inhibition against FLT3, the ionic interaction with Asp829 seems to play a crucial. Compounds incorporating basic moieties (**7b**, **7c**, **7d**, **7e**, **7j**, **7m**, and **7n**) maintained inhibitory activity towards FLT3, while compounds that were substituted with less basic moieties (**7o**, **7p**, and **7q**) lost the activity. The quinazoline derivatives were also tested for their activity against FLT3-ITD, and they displayed inhibitory activity against FLT3-ITD similar to level of activity on FLT3 (**7b**, **7c**, **7d**, **7j, 7m,** and **7n**).

Moreover, the most potent **7d** was examined further for inhibitory activity against other FLT3 mutants and the result was impressive (Table 2). The **7d** was a potent inhibitor towards FLT3 (F594_R_R595) (IC_50_ = 0.524 µM), FLT3 (F594_EY_R595) (IC_50 =_ 0.495 µM), FLT3 (Y591_ VDFREYEYD_V592) (IC_50_ = 0.728 µM), and FLT3 (D835Y), with an IC_50_ value of 0.228 µM. Since FLT3-ITD mutation occur mainly in the juxtamembrane domain away from the ATP binding site, the FLT3 inhibitors binding in ATP site exhibit potency against FLT3-ITD mutant^14^. On the other hand, a mutation in active site such as FLT3 (D835Y) induces active conformation of kinase and type II inhibitors generally could not bind well enough. This is also known to be a mechanism of secondary resistance of type II FLT3 inhibitor.^13^ Nevertheless, our compound **7d**, which is a type II FLT3 inhibitor, exhibits a potent inhibitory activity (IC_50_ = 228 nM) in FLT3-TKD (D835Y) similar to that of FLT3 wild type also. FLT3-TKD, especially D835Y, D835H mutant was identified as a main drug resistance mechanism of FLT3 inhibitor in the clinical trial.^15–18^ Like quizartinib, **7d** displays potent inhibitory activity against FLT3-TKD (D835Y) with value of IC_50_ 228 nM, while decreased about 2-fold compared to that of FLT3 wild type.

**Table 2.** Enzymatic activities of compound **7d** against FLT3 mutants.

Kinase	IC_50_ (µM)
FLT3 (F594_R595 ins R)	0.524
FLT3 (F594_R595 ins EY)	0.495
FLT3 (Y591_V592 ins VDFREYEYD)	0.728
FLT3 (D835Y)	0.228

**Figure 5.**
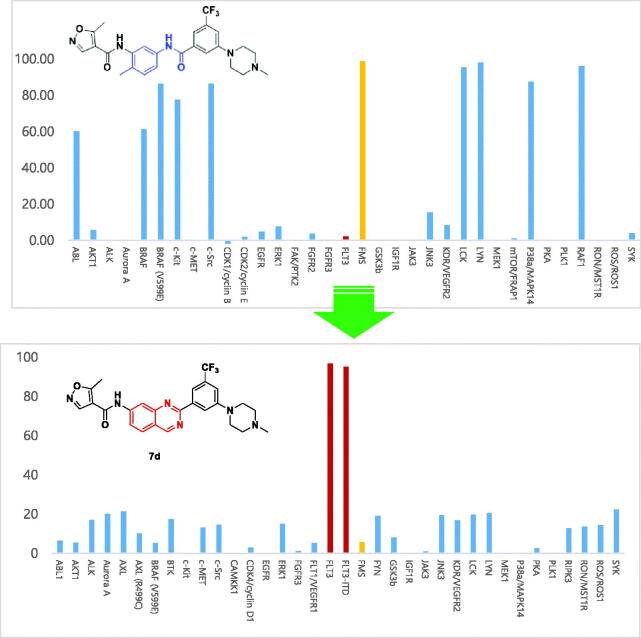
Kinase profiling according to chemical scaffold. The profiles of the FMS kinase inhibitor [13] and quinazoline derivative **7d** (10 μM) are shown.

Next, we investigated kinase panel screening of **7d** and **7b** over 36 different kinases at a single dose of 10 µM (Figure 5). Both compounds **7d** and **7b** (data in Supplementary material) showed an excellent selectivity profile, having almost no activity on other kinases including FMS and cKit. High selectivity over cKit might be an opportunity to avoid myelosuppression toxicity, which was reported from a dual FLT3/cKit inhibitor^19–22^. It is a valuable result to secure such a kinase selectivity profile considering the similarities of the same type III receptor tyrosine kinase (FMS, cKit and FLT3).

**Figure 6.**
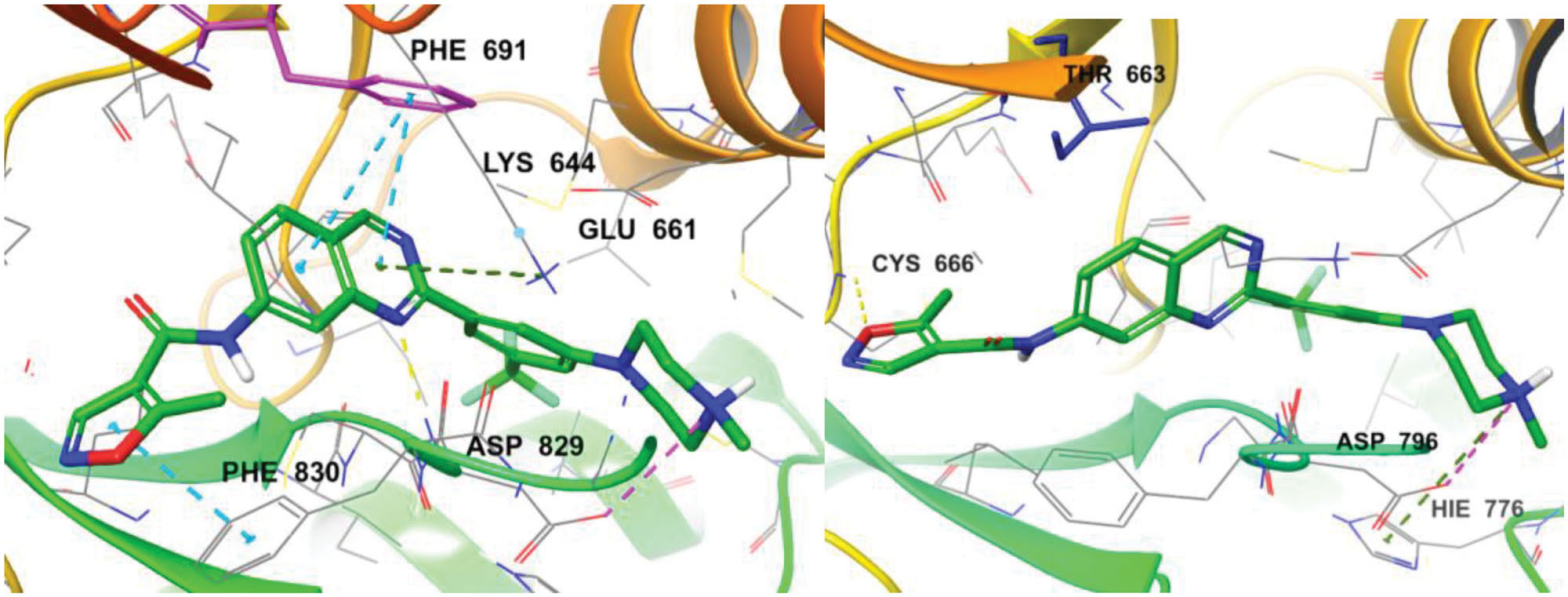
(left) Docking structures of **7d** in FLT3 (PDB: 4RT7) and **7d** in FMS (PDB:3LCO).

For better understanding of the interactions between our compounds and FLT3, molecular docking of compound **7d** into the ATP binding pocket of FLT3 (PDB: 4RT7) was performed using Glide software (Schrödinger, Version 14.2). The docking result is depicted in Figure 6. In the binding model of **7d**, 5-methyisoxazole forms a π-π interaction with Phe830 instead of hydrogen bonding with Cys694, contrast to the co-crystal structure of quirzatinib^23^. Instead, the quinazoline ring of **7d** showed possibilities of multiple interactions: one hydrogen bond with Asp829, π–π interaction with Phe691, and π-cation interaction with Lys644. As it was designed as a hybrid of the middle phenyl ring and urea (or amide) linker, the quinazoline seems tightly bound with ATP-binding site and adjacent region like other type II inhibitors. However, instead of fitting into a hydrophobic pocket surrounded by hydrophobic residue, the strong ionic interaction between the protonated nitrogen of the piperazine ring and the Asp796 might enhance the binding affinity, resulting in potent inhibitory activity and considerable selectivity about FLT3. Due to its strong ionic interaction, the fused ring system of quinazoline might make additional interactions in the active site of FLT3 unlike benzimidazole derivative^12^. The quinazoline derivative **7d** interacts with not only Lys644 (π–cation interaction between guanidine group of Lys644 and quinazoline ring) also gatekeeper residue, Phe691, (π–π interaction between phenyl group of side chain and quinazoline structure) properly, which could contribute to enhanced activity of compound **7d** presumably compared with benzimidazole compound^12^.

We also tried to explain the selectivity of quinazoline compound for FMS and cKit. As shown in Figure 6, FMS kinase has Thr663 instead of Phe691 in the active site. **7d** could not bind well in the FMS kinase without multiple interaction which quinazoline forms. Although **7d** forms hydrogen bonding with Cys666 and strong ionic interaction with Asp796, the quinazoline moiety does not form any interaction with Thr663 or other residue. The difference of only one residue in the highly conserved active site seems to play crucial role in selectivity profile and the interaction between quinazoline structure and Phe691 also seems to be important to binding affinity. In the case of cKit kinase, threonine residue locates at the same position, and same explanation could be applied.

## 3. Conclusions

We designed and synthesised a series of 4-arylamido 5-methylisoxazole derivatives containing quinazoline using a conformational rigidification strategy. Various analogues were synthesised and tested for inhibitory activity against FLT3. Compound **7d** displayed the most potent inhibitory activity against FLT3, with an IC_50_ of 106 nM, and was also active against FLT3-ITD and FLT3-TKD (D835Y), with an IC_50_ of 301 nM and 228 nM respectively. The novel type II FLT3 inhibitors showed an excellent selectivity profile having 20% or less activity towards other kinases including similar FMS, cKit. Specifically, the selectivity over FMS and cKit potentially relieve the myelosuppression toxicity observed in the dual FLT3/cKit kinase inhibitor. Molecular docking of **7d** into the ATP binding site of FLT3 kinase was performed and the result disclosed that the quinazoline structure could perform an adequate or even better bioisostere of benzimidazole in previous report. Considering that FLT3 and FLT3 mutants have been significantly associated with FLT3 mutant-mediated AML, compound **7d** could be a valuable molecule for FLT3-mutant positive physiology and pathology research and a potential therapeutic agent. Even if further optimisation would be necessary in terms of potency, our research towards a novel selective FLT3 inhibitor suggests the potential of quinazoline compounds as AML therapeutics.

## Supplementary Material

Supplemental Material
