# Supplementary material for: Discovery of 5-methyl-N-(2-arylquinazolin-7-yl)isoxazole-4-carboxamide analogues as highly selective FLT3 inhibitors
Source: J Enzyme Inhib Med Chem. 2020 Apr 27;35(1):1110–5. doi: 10.1080/14756366.2020.1758689 (PMC7241567; doi:10.1080/14756366.2020.1758689)
Supplement: Supplemental Material [file IENZ_A_1758689_SM1076.pdf]

Supporting Information

Discovery of 5-methyl-*N*-(2-arylquinazolin-7-yl)isoxazole-4-carboxamide analogues as highly selective FLT3 inhibitors

Daseul Im, Hyungwoo Moon, Jingwoong Kim, Youri Oh, Miyoung Jang, Jung-Mi Hah*

College of Pharmacy and Institute of Pharmaceutical Science and Technology, Hanyang University, 55 Hanyandaehak-ro Sannok-gu, Ansan, Gyeonggi-do, 15588 Korea

*Corresponding author. Tel.: +82-31-400-5803; fax: +82-31-400-5958; e-mail: jhah@hanyang.ac.kr

Contents:

1. Molecular Modelling S2

2. Chemistry..S2

3. Evaluation of IC_{50} and Selected Kinase Profiling..S25

4. Copies of ^{1}H and ^{13}C NMR spectrum of selected compounds S26

5. Percentages of enzymatic inhibition exerted by **7d** and **7b** toward selected protein kinases S35

6. References...S37

1. Molecular Modelling

Compounds were docked into the FLT3 structure (PDB: 4RT7). Protein and ligand preparations were performed with Schrödinger′s tools with standard settings and Glide was used for docking and scoring. The 3D X-ray protein structures of FLT3 wildtype as a complex with a ligand were obtained from the PDB (code: 4RT7) and prepared using the Protein Preparation Wizard of the Schrödinger Maestro program. All water molecules were removed from the structure and it was selected as a template. The structures of inhibitors were drawn using Chemdraw, and their 3D conformation was generated using the Schrödinger LigPrep program with the OPLS 2005 force field. Molecular docking of compound into the structure of FLT-3 wildtype (PDB code: 4RT7) were carried out using Schrodinger Glide (Version 11.5).

2. Chemistry

2.1 General chemical methods

All chemicals were of reagent grade and were purchased from Aldrich (USA). Separation of the compounds by column chromatography was carried out with silica gel 60 (200−300 mesh ASTM, E. Merck, Germany). The quantity of silica gel used was 50−100 times the weight charged on the column. Thin layer chromatography (TLC) was run on the silica gel-coated aluminum sheets (silica gel 60 GF254, E. Merck, Germany) and visualized under ultraviolet (UV) light (254 nm). ^{1}H NMR and ^{13}C NMR spectra were recorded on a Bruker model digital AVANCE III 400 MHz spectrometer at 25 ^{o}C using tetramethylsilane (TMS) as an internal

standard. High-resolution MS (HR/MS) experiments were conducted with a Finnigan LTQ Orbitrap mass spectrometer (Thermo Fisher Scientific Inc, MA, USA) operated in positive-ion electrospray mode.

2.2. 2-amino-4-nitrobenzamide (2)

To a solution of 2-amino-4-nitro-benzoic acid (546.39 mg, 3 mmol) in CH_2Cl_2 (30 mL) was added 1-ethyl-3-(3-dimethylaminopropyl)carbodiimide **(**EDC) (690.12 mg, 3.6 mmol), hydroxybenztriazole (HOBt) (450.42 mg, 3 mmol), trimethylamine (TEA) (0.72 mL), and NH_3 in MeOH (2 M, 40 mL). The reaction was stirred at RT for overnight, and a precipitation formed. The solid was isolated via vacuum filtration. 2-amino-4-nitro-benzamide. (487.8 mg. 89.76%); ^{1}H NMR (400 MHz, DMSO-d_6) δ 8.06 (s, 1H), 7.75 (d, J = 8.6 Hz, 1H), 7.59 (d, J = 1.5 Hz, 1H), 7.51 (s, 1H), 7.31–7.22 (m, 1H), 7.05 (s, 2H).

2.3. 2-(aminomethyl)-5-nitroaniline (3)

Borane-tetrahydrofuran complex (1.0 M solution of tetrahydrofuran) (2.2 mL) was added to a tetrahydrofuran (6.0 mL) solution of 2-amino-4-nitrobenzamide (100.0 mg) and refluxed for 2 hours. The mixture was left to cool. Methanol was then added to the mixture and neutralized with 10% hydrogen chloride in methanol. The solvent was distilled off under reduced pressure. A solution of 1N aqueous sodium hydroxide solution was added to the residue and was extracted with methylene chloride. The organic layer was washed with saturated saline solution and dried over anhydrous sodium sodium sulfate. The solvent was distilled off under reduced pressure. The title crude compound (87.47 mg, 95.13%) was obtained as an orange solid; ^{1}H NMR (400 MHz, DMSO-d_6) δ 7.28–7.22 (m, 1H), 6.48 (s, 2H), 5.73 (dd, J = 7.6, 1.9 Hz, 2H),

5.27 (s, 2H).

2.4. General syntheses of *N*-(2-amino-4-nitrobenzyl)-4-morpholino-3-(trifluoromethyl)benzamide (4a-q)

To dichloromethane (DCM) (3.5 L2-amino-4-nitrobenzylamine (1 eq) and TEA (3.5 eq) were added at 0°C on stirring. Then benzoyl chloride (0.9 eq) in DCM (0.1 M) was added on stirring at such a rate to keep the temperature at 0-5 °C. The mixture was stirred for 3 hours at r.t. Water (1 L) was added and the organic phase was separated, washed with water and dried. Then crude product was purified by flash column chromatography. The title compound was isolated and purified from crude reaction mixture as solid in 91.0% yield; Crude compound was used as a starting material for next step without further purification; HRMS (ESI$^+$) calculated for $C_{19}H_{19}F_3N_4O_4$ $[M+H]^+$: 425.1358, found 425.0768.

_N_-(2-amino-4-nitrobenzyl)-3-morpholino-5-(trifluoromethyl)benzamide (4b)

The title compound was isolated and purified from crude reaction mixture as solid in 70.2% yield; Crude compound was used as a starting material for next step without further purification); HRMS (ESI$^+$) calculated for $C_{19}H_{19}F_3N_4O_4$ $[M+H]^+$: 425.1358, found 425.3289.

_N_-(2-amino-3-nitrophenyl)-3-(4-methyl-1*H*-imidazol-1-yl)-5 (trifluoromethyl) benzamide (4c)

The title compound was isolated and purified from crude reaction mixture as solid in 57.2% yield; ^{1}H NMR (400 MHz, DMSO-d_6) δ 9.34 (t, J = 5.8 Hz, 1H), 8.38 (s, 1H), 8.36 (d, J = 1.3 Hz, 1H), 8.22 (s, 1H), 8.13 (s, 1H), 7.67 (s, 1H), 7.52 (d, J = 2.4 Hz, 1H), 7.37 (dd, J = 8.3, 2.4 Hz, 1H), 7.28 (d, J = 8.4 Hz, 1H), 5.82 (s, 2H), 4.44 (d, J = 5.8 Hz, 2H), 2.18 (d, J = 0.8 Hz,

3H); HRMS (ESI$^+$) calculated for $C_{19}H_{16}F_3N_5O_3$ [M+H]$^+$: 420.1205, found 420.3815.

_N_-(2-amino-4-nitrobenzyl)-3-(4-methylpiperazin-1-yl)-5-(trifluoromethyl)benzamide (4d)

The title compound was isolated and purified from crude reaction mixture as solid in 79.0% yield; ^{1}H NMR (400 MHz, DMSO-d_6) δ 9.19 (t, *J* = 5.9 Hz, 1H), 7.68 (s, 1H), 7.57 (s, 1H), 7.50 (d, *J* = 2.4 Hz, 1H), 7.36 (dd, *J* = 8.3, 2.5 Hz, 2H), 7.23 (d, *J* = 8.3 Hz, 1H), 5.82 (s, 2H), 4.39 (d, *J* = 5.8 Hz, 2H), 3.31–3.27 (m, 4H), 2.48–2.44 (m, 4H), 2.23 (s, 3H); HRMS (ESI$^+$) calculated for $C_{20}H_{22}F_3N_5O_3$ [M+H]$^+$: 438.1675, found 438.3749.

_N_-(2-amino-4-nitrobenzyl)-3-((4-ethylpiperazin-1-yl)methyl)-5-(trifluoromethyl)benzamide (4e)

The title compound was isolated and purified from crude reaction mixture as solid in 67.3% yield; ^{1}H NMR (400 MHz, DMSO-d_6) δ 9.31 (t, *J* = 5.8 Hz, 1H), 8.15 (d, *J* = 5.3 Hz, 2H), 7.85 (s, 1H), 7.52 (d, *J* = 2.4 Hz, 1H), 7.38 (dd, *J* = 8.3, 2.4 Hz, 1H), 7.26 (d, *J* = 8.3 Hz, 1H), 5.84 (s, 2H), 4.42 (d, *J* = 5.8 Hz, 2H), 3.64 (s, 2H), 3.40-3.33 (s, 2H), 3.33–3.29 (m, 4H), 2.45 (s, 4H), 1.02 (t, *J* = 6.9 Hz, 3H); HRMS (ESI$^+$) calculated for $C_{22}H_{26}F_3N_5O_3$ [M+H]$^+$: 466.1988, found 466.5619.

_N_-(2-amino-4-nitrobenzyl)-3-fluoro-5-(trifluoromethyl)benzamide (4f)

The title compound was isolated and purified from crude reaction mixture as solid in 64.8% yield; ^{1}H NMR (400 MHz, DMSO-d_6) δ 9.35 (t, *J* = 5.8 Hz, 1H), 8.12 (d, *J* = 0.6 Hz, 1H), 8.04 (d, *J* = 9.2 Hz, 1H), 7.95 (d, *J* = 8.5 Hz, 1H), 7.51 (d, *J* = 2.4 Hz, 1H), 7.36 (dd, *J* = 8.3, 2.4 Hz, 1H), 7.25 (d, *J* = 8.4 Hz, 1H), 5.80 (s, 2H), 4.41 (d, *J* = 5.8 Hz, 2H); HRMS (ESI$^+$) calculated for $C_{15}H_{11}F_4N_3O_3$ [M+H]$^+$: 358.0737, found 358.0891.

N-(2-amino-4-nitrobenzyl)-3-chloro-4-(trifluoromethyl)benzamide (4g)

The title compound was isolated and purified from crude reaction mixture as solid in 72.7% yield; ^{1}H NMR (400 MHz, DMSO-d_6) δ 9.35 (t, J = 5.8 Hz, 1H), 8.34 (d, J = 2.0 Hz, 1H), 8.20 (dd, J = 8.4, 2.0 Hz, 1H), 7.90 (d, J = 8.4 Hz, 1H), 7.50 (d, J = 2.4 Hz, 1H), 7.35 (dd, J = 8.3, 2.4 Hz, 1H), 7.24 (d, J = 8.4 Hz, 1H), 5.80 (s, 2H), 4.40 (d, J = 5.8 Hz, 2H); HRMS (ESI$^+$) calculated for $C_{15}H_{11}ClF_3N_3O_3$ [M+H]$^+$: 374.0441, found 374.4039.

N-(2-amino-4-nitrobenzyl)-1-phenyl-5-(trifluoromethyl)-1H-pyrazole-4-carboxamide (4h)

The title compound was isolated and purified from crude reaction mixture as solid in 73.3% yield; ^{1}H NMR (400 MHz, DMSO-d_6) δ 9.12 (t, J = 6.0 Hz, 1H), 8.26–8.20 (m, 1H), 7.62–7.56 (m, 3H), 7.52 (dd, J = 6.2, 2.4 Hz, 3H), 7.37 (dd, J = 8.3, 2.4 Hz, 1H), 7.26 (d, J = 8.4 Hz, 1H), 5.80 (s, 2H), 4.36 (d, J = 6.0 Hz, 2H); HRMS (ESI$^+$) calculated for $C_{18}H_{14}F_3N_5O_3$ [M+H]$^+$: 406.1049, found 406.1158.

N-(2-amino-4-nitrobenzyl)-3-chlorobenzamide (4i)

The title compound was isolated and purified from crude reaction mixture as solid in 77.5% yield; ^{1}H NMR (400 MHz, DMSO-d_6) δ 9.16 (t, J = 5.9 Hz, 1H), 7.94 (t, J = 1.8 Hz, 1H), 7.89–7.84 (m, 1H), 7.64 (ddd, J = 8.0, 2.1, 1.1 Hz, 1H), 7.54 (t, J = 7.9 Hz, 1H), 7.50 (d, J = 2.4 Hz, 1H), 7.36 (dd, J = 8.3, 2.4 Hz, 1H), 7.23 (d, J = 8.3 Hz, 1H), 5.81 (s, 2H), 4.37 (d, J = 5.9 Hz, 2H); HRMS (ESI$^+$) calculated for $C_{14}H_{12}ClN_3O_3$ [M+H]$^+$: 306.0567, found 306.2976.

(E)-N-(2-amino-4-nitrobenzyl)-3-(4-methoxyphenyl)acrylamide (4j)

The title compound was isolated and purified from crude reaction mixture as solid in 79.0% yield; ^{1}H NMR (400 MHz, DMSO-d_6) δ 8.58 (s, 1H), 7.58 – 7.53 (m, 2H), 7.50 (d, J = 2.4 Hz,

1H), 7.47 (d, J = 15.8 Hz, 1H), 7.37 (dd, J = 8.3, 2.4 Hz, 1H), 7.23 (d, J = 8.3 Hz, 1H), 7.02 – 6.98 (m, 2H), 6.56 (d, J = 15.8 Hz, 1H), 5.85 (s, 2H), 4.32 (d, J = 6.1 Hz, 2H), 3.81 (s, 3H); HRMS (ESI$^+$) calculated for $C_{17}H_{17}N_3O_4$ $[M+H]^+$: 328.1219, found 328.3400.

(E)-*N*-(2-amino-4-nitrobenzyl)-3-(4-chlorophenyl)acrylamide (4k)

The title compound was isolated and purified from crude reaction mixture as solid in 44.3% yield; ^{1}H NMR (400 MHz, DMSO-d_6) δ 8.67 (t, J = 6.2 Hz, 1H), 7.65–7.58 (m, 2H), 7.53–7.43 (m, 4H), 7.35 (dd, J = 8.3, 2.4 Hz, 1H), 7.22 (d, J = 8.3 Hz, 1H), 6.70 (d, J = 15.8 Hz, 1H), 5.82 (s, 2H), 4.31 (d, J = 6.1 Hz, 2H); HRMS (ESI$^+$) calculated for $C_{16}H_{14}ClN_3O_3$ $[M+H]^+$: 332.0724, found 332.2657.

***N*-(2-amino-4-nitrobenzyl)-5-(tert-butyl)isoxazole-3-carboxamide (4l)**

The title compound was isolated and purified from crude reaction mixture as solid in 37.0% yield; ^{1}H NMR (400 MHz, $CDCl_3$) δ 7.79 (s, 1H), 7.70 (dd, J = 8.2, 1.7 Hz, 1H), 7.47 (s, 1H), 7.38 (d, J = 8.3 Hz, 1H), 6.44 (s, 1H), 4.69 (d, J = 6.5 Hz, 2H), 1.38 (s, 9H); HRMS (ESI$^+$) calculated for $C_{15}H_{18}N_4O_4$ $[M+H]^+$: 319.1328, found 319.4438.

***N*-(2-amino-4-nitrobenzyl)-3-((1-methylpiperidin-4-yl)oxy)-5-(trifluoromethyl)benzamide (4n)**

The title compound was isolated and purified from crude reaction mixture as solid in 32.7% yield; ^{1}H NMR (400 MHz, DMSO-d_6) δ 9.30 (t, J = 5.9 Hz, 1H), 7.83 (s, 1H), 7.79 (s, 1H), 7.52 (t, J = 2.5 Hz, 2H), 7.38 (dd, J = 8.3, 2.4 Hz, 1H), 7.25 (d, J = 8.4 Hz, 1H), 5.83 (d, J = 7.1 Hz, 2H), 4.72 (s, 1H), 4.41 (d, J = 5.8 Hz, 2H), 3.32 (s, 3H), 2.85 (s, 2H), 2.40 (s, 2H), 2.05 (s, 2H), 1.79 (s, 2H); HRMS (ESI$^+$) calculated for $C_{21}H_{23}F_3N_4O_4$ $[M+H]^+$: 453.1671, found 453.2374.

1-acetyl-*N*-(2-amino-4-nitrobenzyl)piperidine-4-carboxamide (4o)

The title compound was isolated and purified from crude reaction mixture as solid in 67.4% yield; ^{1}H NMR (400 MHz, DMSO-d_6) δ 8.39 (t, *J* = 6.0 Hz, 1H), 7.48 (d, *J* = 2.4 Hz, 1H), 7.35 (dd, *J* = 8.3, 2.4 Hz, 1H), 7.16 (d, *J* = 8.3 Hz, 1H), 5.78 – 5.71 (m, 2H), 4.39 – 4.34 (m, 1H), 4.17 (d, *J* = 6.1 Hz, 2H), 3.83 (d, *J* = 13.7 Hz, 1H), 3.04 (dd, *J* = 18.4, 7.6 Hz, 1H), 2.62 – 2.54 (m, 1H), 2.49 – 2.42 (m, 1H), 2.00 (s, 3H), 1.75 (t, *J* = 13.4 Hz, 2H), 1.60 – 1.49 (m, 1H), 1.45 – 1.34 (m, 1H); HRMS (ESI$^+$) calculated for $C_{15}H_{20}N_4O_4$ $[M+H]^+$: 321.1485, found 321.7849.

***N*-(2-amino-4-nitrobenzyl)isonicotinamide (4p)**

The title compound was isolated and purified from crude reaction mixture as solid in 59.7% yield; ^{1}H NMR (400 MHz, DMSO-d_6) δ 9.34 (t, *J* = 5.9 Hz, 1H), 8.77 (dd, *J* = 4.4, 1.7 Hz, 2H), 7.82 (dd, *J* = 4.4, 1.7 Hz, 2H), 7.52 (d, *J* = 2.4 Hz, 1H), 7.38 (dd, *J* = 8.3, 2.4 Hz, 1H), 7.25 (d, *J* = 8.4 Hz, 1H), 5.82 (d, *J* = 7.1 Hz, 2H), 4.41 (d, *J* = 6.0 Hz, 2H); HRMS (ESI$^+$) calculated for $C_{13}H_{12}N_4O_3$ $[M+H]^+$: 273.0909, found 273.3772.

***N*-(2-amino-4-nitrobenzyl)nicotinamide (4q)**

The title compound was isolated and purified from crude reaction mixture as solid in 70.6% yield; ^{1}H NMR (400 MHz, DMSO-d_6) δ 9.46 (t, *J* = 6.3 Hz, 1H), 8.69 (ddd, *J* = 4.8, 1.6, 1.0 Hz, 1H), 8.10–8.05 (m, 1H), 8.05–8.00 (m, 1H), 7.64 (ddd, *J* = 7.3, 4.8, 1.5 Hz, 1H), 7.49 (d, *J* = 2.4 Hz, 1H), 7.35 (dd, *J* = 8.3, 2.4 Hz, 1H), 7.27 (d, *J* = 8.3 Hz, 1H), 5.90 (s, 2H), 4.40 (d, *J* = 6.4 Hz, 2H); HRMS (ESI$^+$) calculated for $C_{13}H_{12}N_4O_3$ $[M+Na]^+$: 295.0802, found 295.3123.

2.5. General syntheses of 4-(3-(7-nitroquinazolin-2-yl)-5-(trifluoromethyl)phenyl)

morpholine (5a-q)

A solution starting material (105.5 mg, 0.2515 mmol) and concd aq HCl (1.2 eq) in acetic acid (0.838 mL) was submitted to microwave irradiation at 150 °C, for 10~30 min until starting material spot disappear in TLC. The reaction mixture was cooled to room temperature, and the product precipitated upon cooling. The precipitate was filtered, washed with acetic acid and *N*, *N*-diisopropylethylamine (DIPEA), and then dried to provide solid as HCl salt. Then after dissolving in ethyl acetate (EA) washed with 1 M aqueous NaOH and dried with Na_2SO_4. free dihydroquinazoline (1 eq) and p-chloranil (1.2 eq) in toluene (0.1 M) are refluxed overnight. The mixture is cooled to room temperature, and the precipitate is filtered off and washed with toluene (350 mL). The filtrate is suspended in 0.5*N* NaOH (400 mL) and the aqueous phase is extracted with dichloromethane (100 mL). The combined organic phases are washed with water and concentrated. The solid is purified by flash column chromatography. The title compound was isolated and purified from crude reaction mixture as solid in 62.3%; ^{1}H NMR (400 MHz, DMSO-d_6) δ 9.98 (d, *J* = 0.8 Hz, 1H), 8.87 (d, *J* = 1.9 Hz, 2H), 8.83 (dd, *J* = 8.5, 2.0 Hz, 1H), 8.50 (dd, *J* = 8.9, 0.5 Hz, 1H), 8.46 (dd, *J* = 8.9, 2.1 Hz, 1H), 7.75 (d, *J* = 8.5 Hz, 1H), 3.81–3.74 (m, 4H), 3.07–2.99 (m, 4H); HRMS (ESI$^+$) calculated for $C_{19}H_{15}F_3N_4O_3$ $[M+H]^+$: 405.1096, found 405.0978.

4-(3-(7-nitroquinazolin-2-yl)-5-(trifluoromethyl)phenyl)morpholine (5b)

The title compound was isolated and purified from crude reaction mixture as solid in 56.9% yield; ^{1}H NMR (400 MHz, DMSO-d_6) δ 9.97 (d, *J* = 0.7 Hz, 1H), 8.89 (d, *J* = 2.1 Hz, 1H), 8.50 (d, *J* = 8.6 Hz, 1H), 8.46 (dd, *J* = 8.9, 2.1 Hz, 1H), 8.40 (s, 1H), 8.27 (s, 1H), 7.45 (s, 1H), 3.85–3.78 (m, 4H), 3.36-3.29 (m, 4H); HRMS (ESI$^+$) calculated for $C_{19}H_{15}F_3N_4O_3$ $[M+H]^+$:

405.1096, found 405.1609.

2-(3-(4-methyl-1*H*-imidazol-1-yl)-5-(trifluoromethyl)phenyl)-7-nitroquinazoline (5c)

The title compound was isolated and purified from crude reaction mixture as solid in 48.2% yield; ^{1}H NMR (400 MHz, DMSO-d_6) δ 10.05 (s, 1H), 8.97 (s, 2H), 8.78 (s, 1H), 8.58–8.48 (m, 3H), 8.29 (s, 1H), 7.77 (s, 1H), 2.24 (s, 3H); HRMS (ESI$^+$) calculated for $C_{19}H_{12}F_3N_5O_2$ [M+H]$^+$: 400.0943, found 400.1503.

2-(3-(4-methylpiperazin-1-yl)-5-(trifluoromethyl)phenyl)-7-nitroquinazoline (5d)

The title compound was isolated and purified from crude reaction mixture as solid in 41.0% yield; ^{1}H NMR (400 MHz, DMSO-d_6) δ 9.96 (d, *J* = 0.8 Hz, 1H), 8.91–8.87 (m, 1H), 8.49 (dd, *J* = 8.9, 0.5 Hz, 1H), 8.45 (dd, *J* = 8.9, 2.1 Hz, 1H), 8.38 (s, 1H), 8.23 (s, 1H), 7.42 (s, 1H), 3.40–3.35 (m, 4H), 2.53 (d, *J* = 5.0 Hz, 4H), 2.26 (s, 3H); HRMS (ESI$^+$) calculated for $C_{20}H_{18}F_3N_5O_2$ [M+H]$^+$: 418.1413, found 418.1756.

2-(3-((4-ethylpiperazin-1-yl)methyl)-5-(trifluoromethyl)phenyl)-7-nitroquinazoline (5e)

The title compound was isolated and purified from crude reaction mixture as solid in 32.8% yield; ^{1}H NMR (400 MHz, $CDCl_3$) δ 9.68 (d, *J* = 0.8 Hz, 1H), 9.05 (d, *J* = 2.2 Hz, 1H), 8.87 (s, 1H), 8.81 (s, 1H), 8.45 (dd, *J* = 8.8, 2.2 Hz, 1H), 8.19 (d, *J* = 8.6 Hz, 1H), 7.85 (s, 1H), 3.75 (s, 2H), 2.63 (s, 4H), 2.54–2.47 (m, 2H), 1.64–1.58 (m, 4H), 1.14 (t, *J* = 7.2 Hz, 3H); HRMS (ESI$^+$) calculated for $C_{22}H_{22}F_3N_5O_2$ [M+H]$^+$: 446.1726, found 446.8992.

2-(3-fluoro-5-(trifluoromethyl)phenyl)-7-nitroquinazoline (5f)

The title compound was isolated and purified from crude reaction mixture as solid in 88.3% yield; ^{1}H NMR (400 MHz, DMSO-d_6) δ 10.02 (d, *J* = 0.8 Hz, 1H), 8.94–8.89 (m, 1H), 8.71 (d, *J* = 0.4 Hz, 1H), 8.60 (d, *J* = 9.6 Hz, 1H), 8.53 (dd, *J* = 8.9, 0.6 Hz, 1H), 8.49 (dd, *J* = 8.9, 2.1

Hz, 1H), 7.99 (d, J = 8.4 Hz, 1H); HRMS (ESI$^+$) calculated for $C_{15}H_7F_4N_3O_2$ [M+H]$^+$: 338.0474, found 338.0284.

2-(4-chloro-3-(trifluoromethyl)phenyl)-7-nitroquinazoline (5g)

The title compound was isolated and purified from crude reaction mixture as solid in 88.5% yield; ^{1}H NMR (400 MHz, $CDCl_3$) δ 9.67 (d, J = 0.8 Hz, 1H), 9.07 (d, J = 2.1 Hz, 1H), 9.03 (dd, J = 1.5, 0.7 Hz, 1H), 8.82 (dd, J = 8.4, 2.1 Hz, 1H), 8.46 (dd, J = 8.9, 2.2 Hz, 1H), 8.22–8.17 (m, 1H), 7.74 (d, J = 8.4 Hz, 1H); HRMS (ESI$^+$) calculated for $C_{15}H_7ClF_3N_3O_2$ [M+H]$^+$: 354.0179, found 354.3435.

7-nitro-2-(1-phenyl-5-(trifluoromethyl)-1*H*-pyrazol-4-yl)quinazoline (5h)

The title compound was isolated and purified from crude reaction mixture as solid in 65.3% yield; ^{1}H NMR (400 MHz, DMSO-d_6) δ 9.97 (d, J = 0.8 Hz, 1H), 8.73 (dt, J = 1.8, 0.8 Hz, 1H), 8.58–8.55 (m, 1H), 8.52–8.47 (m, 2H), 7.63 (s, 5H); HRMS (ESI$^+$) calculated for $C_{18}H_{10}F_3N_5O_2$ [M+H]$^+$: 386.0787, found 386.0962.

2-(3-chlorophenyl)-7-nitroquinazoline (5i)

The title compound was isolated and purified from crude reaction mixture as solid in 78.6% yield; ^{1}H NMR (400 MHz, $CDCl_3$) δ 9.66 (d, J = 0.8 Hz, 1H), 9.01 (d, J = 2.2 Hz, 1H), 8.70 (dd, J = 2.5, 1.2 Hz, 1H), 8.59 (dt, J = 7.2, 1.6 Hz, 1H), 8.43 (dd, J = 8.9, 2.2 Hz, 1H), 8.19–8.15 (m, 1H), 7.57 (dt, J = 8.0, 1.7 Hz, 1H), 7.54 (dd, J = 11.3, 4.0 Hz, 1H); HRMS (ESI$^+$) calculated for $C_{14}H_8ClN_3O_2$ [M+H]$^+$: 286.0305, found 286.2357.

(E)-2-(4-methoxystyryl)-7-nitroquinazoline (5j)

The title compound was isolated and purified from crude reaction mixture as solid in 33.9% yield; ^{1}H NMR (400 MHz, DMSO-d_6) δ 9.81 (s, 1H), 8.69 (d, J = 2.0 Hz, 1H), 8.42 (d, J = 8.8

Hz, 1H), 8.38 (dd, J = 8.9, 2.1 Hz, 1H), 8.19 (d, J = 15.9 Hz, 1H), 7.80 (d, J = 8.7 Hz, 2H), 7.36 (d, J = 15.9 Hz, 1H), 7.04 (d, J = 8.7 Hz, 2H), 3.84 (s, 3H); HRMS (ESI$^+$) calculated for $C_{17}H_{13}N_3O_3$ $[M+H]^+$: 308.0957, found 308.3506.

(E)-2-(4-chlorostyryl)-7-nitroquinazoline (5k)

The title compound was isolated and purified from crude reaction mixture as solid in 59.5% yield; ^1H NMR (400 MHz, DMSO-d_6) δ 9.86 (s, 1H), 8.72 (d, J = 2.0 Hz, 1H), 8.45 (d, J = 8.9 Hz, 1H), 8.42 (dd, J = 8.8, 2.1 Hz, 1H), 8.21 (d, J = 16.0 Hz, 1H), 7.90 (m, 2H), 7.54 (m, 3H); HRMS (ESI$^+$) calculated for $C_{16}H_{10}ClN_3O_2$ $[M+H]^+$: 312.0462, found 312.2404.

5-(tert-butyl)-3-(7-nitroquinazolin-2-yl)isoxazole (5l)

The title compound was isolated and purified from crude reaction mixture as solid in 82.2% yield; ^1H NMR (400 MHz, $CDCl_3$) δ 9.73 (d, J = 0.6 Hz, 1H), 9.08 (dd, J = 1.5, 0.7 Hz, 1H), 8.51 (dd, J = 8.9, 2.2 Hz, 1H), 8.25–8.21 (m, 1H), 6.87 (d, J = 4.5 Hz, 1H), 1.49 (s, 9H); HRMS (ESI$^+$) calculated for $C_{15}H_{14}N_4O_3$ $[M+H]^+$: 299.1066, found 299.0941.

2-(1*H*-indazol-5-yl)-7-nitroquinazoline (5m)

The title compound was isolated and purified from crude reaction mixture as solid in 41.7% yield; ^1H NMR (400 MHz, DMSO-d_6) δ 13.43 (s, 1H), 9.97 (s, 1H), 8.85 (s, 1H), 8.82 (s, 1H), 8.49 (d, J = 8.8 Hz, 1H), 8.44 (dd, J = 8.9, 1.9 Hz, 1H), 8.40 (d, J = 8.5 Hz, 1H), 8.20 (s, 1H), 7.97 (d, J = 8.5 Hz, 1H); HRMS (ESI$^+$) calculated for $C_{15}H_9N_5O_2$ $[M+H]^+$: 292.0756, found 292.3588.

2-(3-((1-methylpiperidin-4-yl)oxy)-5-(trifluoromethyl)phenyl)-7-nitroquinazoline (5n)

The title compound was isolated and purified from crude reaction mixture as solid in 52.8% yield; ^1H NMR (400 MHz, $CDCl_3$) δ 9.67 (d, J = 0.8 Hz, 1H), 9.04 (d, J = 2.2 Hz, 1H), 8.57 (s,

1H), 8.45 (dd, J = 8.9, 2.2 Hz, 1H), 8.43 (s, 1H), 8.19 (d, J = 8.9 Hz, 1H), 7.36 (s, 1H), 4.63 (s, 1H), 2.78 (s, 2H), 2.47 (s, 2H), 2.40 (s, 3H), 2.21 (s, 1H), 2.16 (dd, J = 13.1, 3.7 Hz, 2H), 2.04–1.95 (m, 2H); HRMS (ESI$^+$) calculated for $C_{21}H_{19}F_3N_4O_3$ [M+H]$^+$: 433.1409, found 433.7546.

1-(4-(7-nitroquinazolin-2-yl)piperidin-1-yl)ethan-1-one (5o)

The title compound was isolated and purified from crude reaction mixture as solid in 29.6% yield; ^{1}H NMR (400 MHz, $CDCl_3$) δ 9.55 (d, J = 0.8 Hz, 1H), 8.91 (d, J = 2.2 Hz, 1H), 8.42 (dd, J = 8.9, 2.2 Hz, 1H), 8.16 – 8.12 (m, 1H), 4.82 – 4.74 (m, 1H), 4.03 (d, J = 13.5 Hz, 1H), 3.39 (ddd, J = 15.1, 7.6, 3.6 Hz, 1H), 3.32 (dt, J = 13.6, 4.0 Hz, 1H), 2.87 (td, J = 12.8, 2.9 Hz, 1H), 2.26 – 2.20 (m, 2H), 2.20 (s, 3H), 2.14 – 2.05 (m, 1H), 1.96 (ddd, J = 25.6, 12.1, 4.3 Hz, 1H); HRMS (ESI$^+$) calculated for $C_{15}H_{16}N_4O_3$ [M+H]$^+$: 301.1222, found 301.4713.

7-nitro-2-(pyridin-4-yl)quinazoline (5p)

The title compound was isolated and purified from crude reaction mixture as solid in 10.5% yield; ^{1}H NMR (400 MHz, $CDCl_3$) δ 9.72 (d, J = 0.8 Hz, 1H), 9.05 (dd, J = 1.5, 0.7 Hz, 1H), 8.89 (dd, J = 4.6, 1.5 Hz, 2H), 8.55 (dd, J = 4.5, 1.6 Hz, 2H), 8.49 (dd, J = 8.9, 2.2 Hz, 1H), 8.26–8.20 (m, 1H); HRMS (ESI$^+$) calculated for $C_{13}H_8N_4O_2$ [M+H]$^+$: 253.0647, found 253.2785.

7-nitro-2-(pyridin-2-yl)quinazoline (5q)

The title compound was isolated and purified from crude reaction mixture as solid in 34.5% yield; ^{1}H NMR (400 MHz, $CDCl_3$) δ 9.79 (s, 1H), 9.18 (d, J = 2.1 Hz, 1H), 9.01 (d, J = 4.0 Hz, 1H), 8.80 (d, J = 7.9 Hz, 1H), 8.49 (dd, J = 8.9, 2.2 Hz, 1H), 8.26 – 8.21 (m, 1H), 8.03 (td, J = 7.8, 1.7 Hz, 1H), 7.59 – 7.54 (m, 1H); HRMS (ESI$^+$) calculated for $C_{13}H_8N_4O_2$ [M+H]$^+$: 253.0647, found 253.2424.

2.6. General syntheses of 2-(4-morpholino-3-(trifluoromethyl)phenyl)quinazolin-7-amine (6a-q)

Quinazoline compound (1 eq) and Fe (5 eq) in EtOH/AcOH/H2O = 2:2:1 (0.1 M) was stirred at 60 °C. Stirring was continued for 1*H*and the solution was cooled to room temperature. Solvent was removed in vacuo. Then solution is filtered off by celite using EA or DCM. Extraction was performed by addition of saturated aqueous sodium hydroxide being extracted with ethyl acetate. The organic phase is thoroughly washed with brine, dried over sodium sulfate to produce aniline product as a pure solid. The title compound was isolated and purified from crude reaction mixture as solid in 46.7% yield; ^{1}H NMR (400 MHz, DMSO-d_6) δ 9.15 (d, *J* = 0.6 Hz, 1H), 8.77 (d, *J* = 2.0 Hz, 1H), 8.72 (dd, *J* = 8.4, 1.9 Hz, 1H), 7.78 (d, *J* = 8.8 Hz, 1H), 7.68 (d, *J* = 8.5 Hz, 1H), 7.07 (dd, *J* = 8.8, 2.1 Hz, 1H), 6.87 (d, *J* = 2.0 Hz, 1H), 6.52 (s, 2H), 3.80 – 3.73 (m, 4H), 3.00 – 2.95 (m, 4H); HRMS (ESI$^+$) calculated for $C_{19}H_{17}F_3N_4O$ $[M+H]^+$: 375.1354, found 375.0339.

2-(3-morpholino-5-(trifluoromethyl)phenyl)quinazolin-7-amine (6b)

The title compound was isolated and purified from crude reaction mixture as solid in 83.2% yield; ^{1}H NMR (400 MHz, DMSO-d_6) δ 9.14 (d, *J* = 0.5 Hz, 1H), 8.30 (s, 1H), 8.18 (s, 1H), 7.78 (d, *J* = 8.8 Hz, 1H), 7.35 (s, 1H), 7.06 (dd, *J* = 8.8, 2.1 Hz, 1H), 6.86 (d, *J* = 2.0 Hz, 1H), 6.51 (s, 2H), 3.82–3.78 (m, 4H), 3.32–3.28 (m, 4H); HRMS (ESI$^+$) calculated for $C_{19}H_{17}F_3N_4O$ $[M+H]^+$: 375.1354, found 375.3806.

2-(3-(4-methyl-1*H*-imidazol-1-yl)-5-(trifluoromethyl)phenyl)quinazolin-7-amine (6c)

The title compound was isolated and purified from crude reaction mixture as solid in 100% yield; ^{1}H NMR (400 MHz, $CDCl_3$) δ 9.20 (s, 1H), 8.81 (s, 1H), 8.67 (s, 1H), 8.38 (s, 1H), 8.15 (s, 1H), 7.82 (d, J = 8.7 Hz, 1H), 7.69 (s, 1H), 7.10 (d, J = 8.6 Hz, 1H), 6.90 (s, 1H), 6.59 (s, 2H), 2.20 (s, 3H); HRMS (ESI^{+}) calculated for $C_{19}H_{14}F_3N_5$ $[M+H]^{+}$: 370.1201, found 370.2122.

2-(3-(4-methylpiperazin-1-yl)-5-(trifluoromethyl)phenyl)quinazolin-7-amine (6d)

The title compound was isolated and purified from crude reaction mixture as solid in 79.5% yield; ^{1}H NMR (400 MHz, DMSO-d_6) δ 9.16 (s, 1H), 8.32 (s, 1H), 8.17 (s, 1H), 7.79 (d, J = 8.8 Hz, 1H), 7.34 (s, 1H), 7.08 (dd, J = 8.8, 2.1 Hz, 1H), 6.88 (d, J = 1.9 Hz, 1H), 6.52 (s, 2H), 3.30 (m, 4H), 2.54 (m, 4H), 2.27 (s, 3H); HRMS (ESI^{+}) calculated for $C_{20}H_{20}F_3N_5$ $[M+H]^{+}$: 388.1671, found 388.3958.

2-(3-((4-Ethylpiperazin-1-yl)methyl)-5-(trifluoromethyl)phenyl)quinazolin-7-amine (6e)

The title compound was isolated and purified from crude reaction mixture as solid in 32.8% yield; ^{1}H NMR (400 MHz, DMSO-d_6) δ 9.18 (s, 1H), 8.71 (s, 1H), 8.66 (s, 1H), 7.81 (d, J = 8.8 Hz, 1H), 7.77 (s, 1H), 7.09 (dd, J = 8.8, 2.1 Hz, 1H), 6.89 (d, J = 2.1 Hz, 1H), 6.54 (d, J = 3.7 Hz, 2H), 3.69 (s, 2H), 3.31 (s, 4H), 2.46 (s, 4H), 2.36–2.30 (m, 2H), 1.00 (t, J = 7.2 Hz, 3H); HRMS (ESI^{+}) calculated for $C_{22}H_{24}F_3N_5$ $[M+H]^{+}$: 416.1984, found 416.8992.

2-(3-Fluoro-5-(trifluoromethyl)phenyl)quinazolin-7-amine (6f)

The title compound was isolated and purified from crude reaction mixture as solid in 89.4% yield; ^{1}H NMR (400 MHz, DMSO-d_6) δ 9.17 (d, J = 0.6 Hz, 1H), 8.61 (s, 1H), 8.47 (d, J = 10.0 Hz, 1H), 7.85 (d, J = 8.4 Hz, 1H), 7.81 (d, J = 8.8 Hz, 1H), 7.10 (dd, J = 8.8, 2.1 Hz, 1H), 6.88 (d, J = 2.0 Hz, 1H), 6.58 (s, 2H); HRMS (ESI^{+}) calculated for $C_{15}H_9F_4N_3$ $[M+H]^{+}$: 308.0733, found 308.3506.

2-(4-Chloro-3-(trifluoromethyl)phenyl)quinazolin-7-amine (6g)

The title compound was isolated and purified from crude reaction mixture as solid in 96.3% yield; ^{1}H NMR (400 MHz, DMSO-d_6) δ 9.16 (d, J = 0.6 Hz, 1H), 8.87 (d, J = 2.0 Hz, 1H), 8.73 (dd, J = 8.5, 2.0 Hz, 1H), 7.89 (d, J = 8.5 Hz, 1H), 7.79 (d, J = 8.8 Hz, 1H), 7.08 (dd, J = 8.8, 2.1 Hz, 1H), 6.87 (d, J = 2.1 Hz, 1H), 6.56 (s, 2H); HRMS (ESI$^+$) calculated for $C_{15}H_9ClF_3N_3$ $[M+H]^+$: 324.0437, found 324.1620.

2-(1-Phenyl-5-(trifluoromethyl)-1*H*-pyrazol-4-yl)quinazolin-7-amine (6h)

The title compound was isolated and purified from crude reaction mixture as solid in 86.1% yield; ^{1}H NMR (400 MHz, DMSO-d_6) δ 9.11 (d, J = 0.6 Hz, 1H), 8.36 (s, 1H), 7.77 (d, J = 8.8 Hz, 1H), 7.60 (d, J = 1.7 Hz, 5H), 7.07 (dd, J = 8.8, 2.1 Hz, 1H), 6.81 (d, J = 2.1 Hz, 1H), 6.50 (s, 2H); HRMS (ESI$^+$) calculated for $C_{18}H_{12}F_3N_5$ $[M+H]^+$: 356.1045, found 356.2275.

2-(3-Chlorophenyl)quinazolin-7-amine (6i)

The title compound was isolated and purified from crude reaction mixture as solid in 98.3% yield; ^{1}H NMR (400 MHz, DMSO-d_6) δ 9.15 (d, J = 0.6 Hz, 1H), 8.49–8.42 (m, 2H), 7.79 (d, J = 8.8 Hz, 1H), 7.62–7.54 (m, 2H), 7.08 (dd, J = 8.8, 2.1 Hz, 1H), 6.86 (d, J = 2.1 Hz, 1H), 6.52 (s, 2H); HRMS (ESI$^+$) calculated for $C_{14}H_{10}ClN_3$ $[M+H]^+$: 256.0563, found 256.0520.

(E)-2-(4-methoxystyryl)quinazolin-7-amine (6j)

The title compound was isolated and purified from crude reaction mixture as solid in 79.6% yield; ^{1}H NMR (400 MHz, DMSO-d_6) δ 8.99 (s, 1H), 7.92 (d, J = 16.0 Hz, 1H), 7.68 (dd, J = 8.8, 2.2 Hz, 3H), 7.12 (d, J = 16.0 Hz, 1H), 6.98 (dd, J = 8.6, 6.2 Hz, 3H), 6.74 (d, J = 2.0 Hz, 1H), 6.35 (s, 2H), 3.80 (s, 3H); HRMS (ESI$^+$) calculated for $C_{17}H_{15}N_3O$ $[M+H]^+$: 278.1215, found 278.3117.

(E)-2-(4-chlorostyryl)quinazolin-7-amine (6k)

The title compound was isolated and purified from crude reaction mixture as solid in 83.4% yield; ^{1}H NMR (400 MHz, DMSO-d_6) δ 9.04 (s, 1H), 7.96 (d, J = 16.0 Hz, 1H), 7.79 (d, J = 8.4 Hz, 2H), 7.73 (d, J = 8.7 Hz, 1H), 7.49 (d, J = 8.4 Hz, 2H), 7.30 (d, J = 16.0 Hz, 1H), 7.07–6.98 (m, 1H), 6.78 (s, 1H), 6.41 (s, 2H); HRMS (ESI$^+$) calculated for $C_{16}H_{12}ClN_3$ [M+H]$^+$: 282.0720, found 282.2737.

2-(5-(tert-Butyl)isoxazol-3-yl)quinazolin-7-amine (6l)

The title compound was isolated and purified from crude reaction mixture as solid in 87.8% yield; ^{1}H NMR (400 MHz, DMSO-d_6) δ 9.13 (s, 1H), 7.80 (d, J = 8.8 Hz, 1H), 7.11 (dd, J = 8.8, 2.1 Hz, 1H), 6.84 (d, J = 2.0 Hz, 1H), 6.77 (s, 1H), 6.58 (s, 2H), 1.38 (s, 9H); HRMS (ESI$^+$) calculated for $C_{15}H_{16}N_4O$ [M+H]$^+$: 269.1324, found 269.1630.

2-(1*H*-indazol-5-yl)quinazolin-7-amine (6m)

The title compound was isolated and purified from crude reaction mixture as solid in 74.3% yield; ^{1}H NMR (400 MHz, DMSO-d_6) δ 13.25 (s, 1H), 9.16 (s, 1H), 8.69 (d, J = 0.9 Hz, 1H), 8.31 (dd, J = 8.5, 1.3 Hz, 1H), 8.14 (d, J = 1.1 Hz, 1H), 7.87 (d, J = 8.5 Hz, 1H), 7.78 (d, J = 8.7 Hz, 1H), 7.05 (dd, J = 8.7, 2.1 Hz, 1H), 6.88 (d, J = 2.1 Hz, 1H), 6.45 (d, J = 4.1 Hz, 2H); HRMS (ESI$^+$) calculated for $C_{15}H_{11}N_5$ [M+H]$^+$: 262.1014, found 262.3553.

2-(3-((1-Methylpiperidin-4-yl)oxy)-5-(trifluoromethyl)phenyl)quinazolin-7-amine (6n)

The title compound was isolated and purified from crude reaction mixture as solid in 99.8% yield ^{1}H NMR (400 MHz, DMSO-d_6) δ 9.17 (s, 1H), 8.34 (s, 1H), 8.28 (s, 1H), 7.80 (d, J = 8.8 Hz, 1H), 7.41 (s, 1H), 7.09 (dd, J = 8.8, 2.1 Hz, 1H), 6.88 (d, J = 2.0 Hz, 1H), 6.54 (s, 2H), 4.69 – 4.61 (m, 1H), 2.63 (d, J = 5.6 Hz, 2H), 2.27 (t, J = 8.7 Hz, 2H), 2.22 (s, 3H), 1.99 (s, 2H), 1.79 – 1.68 (m, 2H); HRMS (ESI$^+$) calculated for $C_{21}H_{21}F_3N_4O$ [M+H]$^+$: 403.1667, found 403.5034.

1-(4-(7-Aminoquinazolin-2-yl)piperidin-1-yl)ethan-1-one (6o)

The title compound was isolated and purified from crude reaction mixture as solid in 83.0% yield; ^{1}H NMR (400 MHz, DMSO-d_6) δ 8.98 (s, 1H), 7.70 (t, *J* = 6.4 Hz, 1H), 7.00 (dd, *J* = 8.8, 2.1 Hz, 1H), 6.72 (d, *J* = 2.1 Hz, 1H), 6.36 (s, 2H), 4.46 (d, *J* = 13.1 Hz, 1H), 3.92 (d, *J* = 13.6 Hz, 1H), 3.24 – 3.16 (m, 1H), 3.05 (tt, *J* = 11.4, 3.8 Hz, 1H), 2.75 – 2.67 (m, 1H), 2.06 – 2.03 (m, 3H), 1.96 (dd, *J* = 24.1, 7.7 Hz, 2H), 1.80 (ddd, *J* = 25.0, 12.5, 4.3 Hz, 1H), 1.64 (ddd, *J* = 16.5, 12.6, 4.4 Hz, 1H); HRMS (ESI$^+$) calculated for $C_{15}H_{18}N_4O$ $[M+H]^+$: 271.1481, found 271.4682.

2-(Pyridin-4-yl)quinazolin-7-amine (6p)

The title compound was isolated and purified from crude reaction mixture as solid in 96.0% yield; ^{1}H NMR (400 MHz, DMSO-d_6) δ 9.20 (d, *J* = 0.6 Hz, 1H), 8.76 (dd, *J* = 4.5, 1.6 Hz, 2H), 8.35 (dd, *J* = 4.5, 1.6 Hz, 2H), 7.82 (d, *J* = 8.8 Hz, 1H), 7.12 (dd, *J* = 8.8, 2.1 Hz, 1H), 6.90 (d, *J* = 2.1 Hz, 1H), 6.58 (s, 2H); HRMS (ESI$^+$) calculated for $C_{13}H_{10}N_4$ $[M+H]^+$: 223.0905, found 223.3095.

2-(Pyridin-2-yl)quinazolin-7-amine (6q)

The title compound was isolated and purified from crude reaction mixture as solid in 84.12% yield; ^{1}H NMR (400 MHz, $CDCl_3$) δ 9.26 (d, *J* = 0.6 Hz, 1H), 8.90 (ddd, *J* = 4.8, 1.8, 0.9 Hz, 1H), 8.65 (dt, *J* = 8.0, 1.0 Hz, 1H), 7.90 (td, *J* = 7.8, 1.8 Hz, 1H), 7.76 (d, *J* = 8.7 Hz, 1H), 7.42 (ddd, *J* = 7.5, 4.8, 1.2 Hz, 1H), 7.25 (d, *J* = 2.2 Hz, 1H), 7.04 (dd, *J* = 8.7, 2.2 Hz, 1H), 4.46 (d, *J* = 14.2 Hz, 2H); HRMS (ESI$^+$) calculated for $C_{13}H_{10}N_4$ $[M+H]^+$: 223.0905, found 223.1655.

2.6. 5-Methylisoxazole-4-carbonyl chloride

The 5-methylisoxazole-4-carboxylic acid (1 g, 7.86 mmol) in $SOCl_2$ (3 mL) was heated at 50 °C until compound acid disappeared in TLC. After reaction termination, the mixture was cooled to ambient temperature and solvent was evaporated under reduced pressure. 5-

methylisoxazole-4-carbonyl chloride as a crude yellow oil (96%) was used for the next step without further purification; ^{1}H NMR (400 MHz, DMSO-d_6) δ 8.77 (1H, s), 2.64 (3H, s).

2.7. General syntheses of 5-methyl-*N*-(2-(3-morpholino-5-(trifluoromethyl)phenyl) quinazolin-7-yl)isoxazole-4-carboxamide (7a-q)

The mixture of **5**-methylisoxazole-4-carbonyl chloride (1.2 eq~2 eq), aniline compound (1 eq), DIPEA (1 eq) in THF or DCM (0.1 M) was stirred at room temperature until aniline compound disappeared in TLC. After completion of the reaction, the mixture was cooled to ambient temperature and solvent was removed in vacuo. The reaction mixture diluted with ethyl acetate and washed with saturated aqueous sodium bicarbonate. The organic layer dried over $MgSO_4$. The concentrated crude product was purified by flash column chromatography to afford Desired product as a pure solid. The title compound was isolated and purified from crude reaction mixture as solid in 32.5% yield; ^{1}H NMR (400 MHz, , DMSO-d_6) δ 10.54 (s, 1H), 9.56 (d, *J* = 0.7 Hz, 1H), 9.16 (d, *J* = 0.6 Hz, 1H), 8.82 (d, *J* = 2.0 Hz, 1H), 8.77 (dd, *J* = 8.5, 1.9 Hz, 1H), 8.59 (d, *J* = 1.9 Hz, 1H), 8.15 (d, *J* = 8.8 Hz, 1H), 7.90 (dd, *J* = 8.8, 2.0 Hz, 1H), 7.69 (d, *J* = 8.5 Hz, 1H), 3.79 – 3.75 (m, 4H), 3.02 – 2.97 (m, 4H), 2.75 (d, *J* = 0.4 Hz, 3H); ^{13}C NMR (101 MHz, , DMSO-d_6) δ 173.6, 160.2, 159.9, 158.6, 153.7, 150.9, 149.0, 144.1, 133.6, 132.8, 128.5, 125.5, 125.0, 124.2, 122.8, 121.7, 120.1, 115.0, 111.8, 66.5, 53.2, 12.2; HRMS (ESI+) calculated for $C_{24}H_{20}F_3N_5O_3$ [M+H]+: 484.1518, found 484.4126..

5-Methyl-*N*-(2-(3-morpholino-5-(trifluoromethyl)phenyl)quinazolin-7-yl)isoxazole-4-carboxamide (7b)

The title compound was isolated and purified from crude reaction mixture as solid in 53.2%

yield; ^{1}H NMR (400 MHz, DMSO-d_6) δ 10.56 (s, 1H), 9.59 (d, J = 0.7 Hz, 1H), 9.16 (d, J = 0.6 Hz, 1H), 8.67 (d, J = 1.9 Hz, 1H), 8.38 (s, 1H), 8.26 (s, 1H), 8.18 (d, J = 8.8 Hz, 1H), 7.90 (dd, J = 8.8, 2.0 Hz, 1H), 7.41 (s, 1H), 3.87–3.74 (m, 4H), 3.34 (d, J = 4.9 Hz, 4H), 2.74 (s, 3H); ^{13}C NMR (101 MHz, $CDCl_3$) δ 174.5, 160.2, 160.0, 159.4, 151.7 151.6, 148.1, 143.2, 139.6, 132.0, 131.7, 128.0, 125.7, 121.7, 120.9, 118.2, 116.7, 113.6, 111.9, 66.8, 48.9, 12.8; HRMS (ESI$^+$) calculated for $C_{24}H_{20}F_3N_5O_3$ [M+H]$^+$: 484.1518, found 484.3579.

5-Methyl-*N*-(2-(3-(4-methyl-1*H*-imidazol-1-yl)-5-(trifluoromethyl)phenyl) quinazolin-7-yl)isoxazole-4-carboxamide (7c)

The title compound was isolated and purified from crude reaction mixture as solid in 11.1% yield; ^{1}H NMR (400 MHz, DMSO-d_6) δ 10.60 (s, 1H), 9.65 (s, 1H), 9.17 (s, 1H), 8.89 (s, 1H), 8.74 (s, 1H), 8.72 (s, 1H), 8.42 (s, 1H), 8.23 (d, 1H), 8.21 (s, 1H), 7.93 (dd, J = 8.8, 1.8 Hz, 1H), 7.73 (s, 1H), 2.75 (s, 3H), 2.21 (s, 3H); ^{13}C NMR (101 MHz, DMSO-d_6) δ 174.2, 161.5, 160.9, 160.5, 160.5, 151.4, 149.6, 149.5, 144.8, 141.0, 139.6, 138.8, 137.3, 137.0, 136.1, 136.1, 123.1, 122.9, 122.8, 122.2, 121.0, 112.2, 67.3, 12.7; HRMS (ESI$^+$) calculated for $C_{24}H_{17}F_3N_6O_2$ [M+H]$^+$: 479.1365, found 479.3079.

5-Methyl-*N*-(2-(3-(4-methylpiperazin-1-yl)-5-(trifluoromethyl)phenyl)quinazolin-7-yl)isoxazole-4-carboxamide (7d)

The title compound was isolated and purified from crude reaction mixture as solid in 3.33% yield; ^{1}H NMR (400 MHz, DMSO-d_6) δ 10.60 (s, 1H), 9.60 (s, 1H), 9.19 (s, 1H), 8.69 (s, 1H), 8.39 (s, 1H), 8.25 (s, 1H), 8.20 (d, J = 8.8 Hz, 1H), 7.93 (dd, J = 8.9, 1.9 Hz, 1H), 7.42 (s, 1H), 3.42 (s, 4H), 2.76 (s, 3H), 2.67 (s, 4H), 2.37 (s, 3H); ^{13}C NMR (101 MHz, DMSO-d_6) δ 174.2, 160.6, 160.5, 159.5, 152.0, 151.4, 149.6, 149.6, 144.6, 139.8, 130.8, 129.1, 122.4, 122.4, 120.9, 120.9, 117.8, 115.6, 112.3, 54.6, 47.7, 47.7, 12.8; HRMS (ESI$^+$) calculated for $C_{25}H_{23}F_3N_6O_2$ [M+H]$^+$: 497.1835, found 497.3947.

***N*-(2-(3-((4-ethylpiperazin-1-yl)methyl)-5-(trifluoromethyl)phenyl)quinazolin-7-yl)-5-methylisoxazole-4-carboxamide (7e)**

The title compound was isolated and purified from crude reaction mixture as solid in 12.3% yield; ^{1}H NMR (400 MHz, $CDCl_3$) δ 9.38 (s, 1H), 8.80 (s, 1H), 8.73 (s, 2H), 8.32 (s, 2H), 8.02 (d, J = 7.6 Hz, 1H), 7.96 (d, J = 8.8 Hz, 1H), 7.77 (s, 1H), 3.75 (s, 2H), 2.86 (s, 3H), 2.72 (s, 4H), 2.64 (d, J = 7.2 Hz, 2H), 1.97 (s, 4H), 1.19 (d, J = 7.2 Hz, 3H); HRMS (ESI$^+$) calculated for $C_{27}H_{27}F_3N_6O_2$ [M+H]$^+$: 525.2148, found 525.6202.

***N*-(2-(3-fluoro-5-(trifluoromethyl)phenyl)quinazolin-7-yl)-5-methylisoxazole-4-carboxamide (7f)**

The title compound was isolated and purified from crude reaction mixture as solid in 94.7% yield; ^{1}H NMR (400 MHz, $CDCl_3$) δ 9.34 (s, 1H), 9.15 (s, 1H), 8.93 (s, 1H), 8.72 (s, 1H), 8.50 (d, J = 9.9 Hz, 1H), 8.43 (s, 1H), 8.05 (d, J = 7.8 Hz, 1H), 7.90 (d, J = 8.7 Hz, 1H), 7.46 (d, J = 8.0 Hz, 1H), 2.83 (s, 3H); ^{13}C NMR (101 MHz, $CDCl_3$) δ 174.6, 164.0, 161.6, 160.0, 159.6, 158.6, 151.3, 148.2, 143.6, 141.3, 141.2, 128.1, 122.3, 121.2, 120.9, 118.8, 118.6, 116.5, 111.9, 12.8; HRMS (ESI$^+$) calculated for $C_{20}H_{12}F_4N_4O_2$ [M+H]$^+$: 417.0896, found 417.3687.

***N*-(2-(4-chloro-3-(trifluoromethyl)phenyl)quinazolin-7-yl)-5-methylisoxazole-4-carboxamide (7g)**

The title compound was isolated and purified from crude reaction mixture as solid in 80.4% yield; ^{1}H NMR (400 MHz, DMSO-d_6) δ 10.58 (s, 1H), 9.60 (s, 1H), 9.16 (s, 1H), 8.92 (d, J = 1.8 Hz, 1H), 8.79 (dd, J = 8.4, 1.8 Hz, 1H), 8.62 (d, J = 1.6 Hz, 1H), 8.18 (d, J = 8.8 Hz, 1H), 7.99–7.88 (m, 2H), 2.74 (s, 3H); ^{13}C NMR (101 MHz, DMSO-d_6) δ 174.2, 170.8, 160.9, 160.5, 151.3, 149.5, 144.8, 137.5, 135.1, 133.6, 133.6, 132.9, 132.9, 132.9, 129.2, 122.7, 120.9, 115.5, 112.3, 12.8; HRMS (ESI$^+$) calculated for $C_{20}H_{12}ClF_3N_4O_2$ [M+H]$^+$: 433.0601, found 433.1064.

5-Methyl-*N*-(2-(1-phenyl-5-(trifluoromethyl)-1*H*-pyrazol-4-yl)quinazolin-7-

yl)isoxazole-4-carboxamide (7h)

The title compound was isolated and purified from crude reaction mixture as solid in 80.4% yield; ^{1}H NMR (400 MHz, DMSO-d_6) δ 10.57 (s, 1H), 9.58 (d, *J* = 0.7 Hz, 1H), 9.16 (d, *J* = 0.6 Hz, 1H), 8.52 (d, *J* = 2.0 Hz, 1H), 8.49 (s, 1H), 8.18 (d, *J* = 8.8 Hz, 1H), 8.01 (dd, *J* = 8.9, 2.0 Hz, 1H), 7.61 (d, *J* = 7.5 Hz, 5H), 2.78–2.71 (m, 3H); ^{13}C NMR (101 MHz, DMSO-d_6) δ 174.3, 174.2, 163.1, 160.4, 151.3, 151.2, 149.6, 144.7, 142.3, 140.1, 130.2, 129.8, 129.0, 126.4, 125.6, 122.5, 121.7, 120.2, 115.3, 112.2, 12.8; HRMS (ESI$^+$) calculated for $C_{23}H_{15}F_3N_6O_2$ $[M+H]^+$: 465.1209, found 465.3015.

***N*-(2-(3-chlorophenyl)quinazolin-7-yl)-5-methylisoxazole-4-carboxamide (7i)**

The title compound was isolated and purified from crude reaction mixture as solid in 95.1% yield; ^{1}H NMR (400 MHz, DMSO-d_6) δ 10.57 (s, 1H), 9.56 (d, *J* = 0.7 Hz, 1H), 9.16 (d, *J* = 0.6 Hz, 1H), 8.59 (d, *J* = 2.0 Hz, 1H), 8.50 (dtd, *J* = 8.3, 2.4, 1.6 Hz, 2H), 8.15 (d, *J* = 8.8 Hz, 1H), 7.92 (dd, *J* = 8.8, 2.0 Hz, 1H), 7.63–7.57 (m, 2H), 2.74 (d, *J* = 0.5 Hz, 3H); ^{13}C NMR (101 MHz, DMSO-d_6) δ 174.1, 170.8, 160.7, 160.4, 159.3, 151.4, 149.6, 144.6, 140.2, 134.1, 131.2, 131.0, 129.0, 128.0, 122.4, 120.8, 115.6, 112.3, 12.73; HRMS (ESI$^+$) calculated for $C_{19}H_{13}ClN_4O_2$ $[M+H]^+$: 365.0727, found 365.6163.

(E)-*N*-(2-(4-methoxystyryl)quinazolin-7-yl)-5-methylisoxazole-4-carboxamide (7j)

The title compound was isolated and purified from crude reaction mixture as solid in 95.1% yield; ^{1}H NMR (400 MHz, DMSO-d_6) δ 10.54 (s, 1H), 9.44 (s, 1H), 9.16 (d, *J* = 0.6 Hz, 1H), 8.46 (d, *J* = 1.9 Hz, 1H), 8.09 (dd, *J* = 12.4, 8.8 Hz, 2H), 7.88 (dd, *J* = 8.8, 2.0 Hz, 1H), 7.76 (d, *J* = 8.7 Hz, 2H), 7.28 (d, *J* = 16.0 Hz, 1H), 7.03 (d, *J* = 8.8 Hz, 2H), 3.83 (s, 3H), 2.78 – 2.73 (m, 3H); ^{13}C NMR (101 MHz, DMSO-d_6) δ 174.0, 169.0, 161.8, 160.7, 160.4, 160.0, 151.5, 149.6, 144.2, 138.0, 129.8, 128.9, 126.1, 121.5, 120.2, 115.3, 114.9, 112.4, 55.8, 12.7; HRMS (ESI$^+$) calculated for $C_{22}H_{18}N_4O_3$ $[M+H]^+$: 387.1379, found 387.2971.

(E)-*N*-(2-(4-chlorostyryl)quinazolin-7-yl)-5-methylisoxazole-4-carboxamide (7k)

The title compound was isolated and purified from crude reaction mixture as solid in 72.5% yield; ^{1}H NMR (400 MHz, DMSO-d_6) δ 10.54 (s, 1H), 9.46 (s, 1H), 9.14 (d, *J* = 0.6 Hz, 1H), 8.48 (d, *J* = 2.0 Hz, 1H), 8.10 (dd, *J* = 12.4, 9.7 Hz, 2H), 7.89 (dd, *J* = 8.8, 2.0 Hz, 1H), 7.84 (d, *J* = 8.5 Hz, 2H), 7.53 – 7.47 (m, 2H), 7.43 (d, *J* = 16.0 Hz, 1H), 2.73 (d, *J* = 0.4 Hz, 3H); ^{13}C NMR (101 MHz, DMSO-d_6) δ 174.0, 161.2, 160.4, 160.0, 151.4, 149.5, 144.3, 136.7, 135.2, 134.0, 129.8, 129.4, 129.3, 128.9, 121.8, 120.3, 115.4, 112.3, 12.7; HRMS (ESI$^+$) calculated for $C_{21}H_{15}ClN_4O_2$ [M+H]$^+$: 391.0884, found 391.2947.

***N*-(2-(5-(tert-butyl)isoxazol-3-yl)quinazolin-7-yl)-5-methylisoxazole-4-carboxamide (7l)**

The title compound was isolated and purified from crude reaction mixture as solid in 59.2% yield; ^{1}H NMR (400 MHz, DMSO-d_6) δ 10.59 (s, 1H), 9.58 (s, 1H), 9.14 (s, 1H), 8.62 (s, 1H), 8.19 (d, *J* = 8.1 Hz, 1H), 7.95 (d, *J* = 8.0 Hz, 1H), 6.89 (s, 1H), 2.73 (s, 3H), 1.39 (s, 9H); ^{13}C NMR (101 MHz, $CDCl_3$) δ 182.7, 174.4, 162.7, 160.3, 159.7, 154.6, 151.3, 148.5, 144.5, 128.0, 123.1, 121.2, 116.3, 112.0, 99.0, 33.0, 28.8, 12.8; HRMS (ESI$^+$) calculated for $C_{20}H_{19}N_5O_3$ [M+H]$^+$: 378.1488, found 378.4376.

***N*-(2-(1*H*-indazol-5-yl)quinazolin-7-yl)-5-methylisoxazole-4-carboxamide (7m)**

The title compound was isolated and purified from crude reaction mixture as solid in 7.6% yield; ^{1}H NMR (400 MHz, DMSO-d_6) δ 13.34 (s, 1H), 10.59 (s, 1H), 9.61 (s, 1H), 9.18 (s, 1H), 8.78 (d, *J* = 0.6 Hz, 1H), 8.62 (d, *J* = 1.5 Hz, 1H), 8.39 (dd, *J* = 8.6, 1.1 Hz, 1H), 8.20–8.15 (m, 2H), 7.97–7.91 (m, 2H), 2.77 (s, 3H); HRMS (ESI$^+$) calculated for $C_{20}H_{14}N_6O_2$ [M+H]$^+$: 371.1178, found 371.4147.

5-Methyl-*N*-(2-(3-((1-methylpiperidin-4-yl)oxy)-5-(trifluoromethyl)phenyl)quinazolin-7-yl)isoxazole-4-carboxamide (7n)

The title compound was isolated and purified from crude reaction mixture as solid in 3.5%

yield; ^{1}H NMR (400 MHz, $CDCl_3$) δ 9.36 (s, 1H), 8.93 (s, 1H), 8.69 (s, 1H), 8.50 (s, 1H), 8.44 (d, *J* = 1.0 Hz, 1H), 8.32 (s, 1H), 8.14 (dd, *J* = 8.6, 1.1 Hz, 1H), 7.98 (d, *J* = 8.9 Hz, 1H), 7.25 (s, 1H), 4.83 (m, 1H), 3.11 (m, 2H), 2.86 (s, 3H), 2.46 (d, *J* = 20.9 Hz, 2H), 2.20 (d, *J* = 8.2 Hz, 2H), 2.06 (d, *J* = 6.9 Hz, 2H), 1.26 (s, 3H); HRMS (ESI$^+$) calculated for $C_{26}H_{24}F_3N_5O_3$ [M+H]$^+$: 512.1831, found 512.6204.

N-(2-(1-acetylpiperidin-4-yl)quinazolin-7-yl)-5-methylisoxazole-4-carboxamide (7o)

The title compound was isolated and purified from crude reaction mixture as solid in 30.3% yield; ^{1}H NMR (400 MHz, DMSO-d_6) δ 10.55 (s, 1H), 9.44 (s, 1H), 9.16 (s, 1H), 8.47 (d, *J* = 1.6 Hz, 1H), 8.11 (d, *J* = 8.8 Hz, 1H), 7.90 (dd, *J* = 8.8, 1.9 Hz, 1H), 4.48 (d, *J* = 13.0 Hz, 1H), 3.95 (d, *J* = 13.5 Hz, 1H), 3.28 – 3.20 (m, 2H), 2.81 – 2.72 (m, 4H), 2.12 – 2.01 (m, 5H), 1.86 (ddd, *J* = 16.0, 12.4, 4.2 Hz, 1H), 1.69 (ddd, *J* = 16.1, 12.5, 4.1 Hz, 1H); HRMS (ESI$^+$) calculated for $C_{20}H_{21}N_5O_3$ [M+H]$^+$: 380.1644, found 380.5264.

5-Methyl-N-(2-(pyridin-4-yl)quinazolin-7-yl)isoxazole-4-carboxamide (7p)

The title compound was isolated and purified from crude reaction mixture as solid in 50.6% yield; ^{1}H NMR (400 MHz, DMSO-d_6) δ 10.65 (s, 1H), 9.67 (s, 1H), 9.19 (s, 1H), 8.83 (d, *J* = 6.0 Hz, 2H), 8.68 (s, 1H), 8.44 (dd, *J* = 4.5, 1.6 Hz, 2H), 8.24 (d, *J* = 8.8 Hz, 1H), 8.01 (dd, *J* = 8.8, 1.9 Hz, 1H), 2.76 (s, 3H); HRMS (ESI$^+$) calculated for $C_{18}H_{13}N_5O_2$ [M+H]$^+$: 332.1069, found 332.3738.

5-Methyl-N-(2-(pyridin-2-yl)quinazolin-7-yl)isoxazole-4-carboxamide (7q)

The title compound was isolated and purified from crude reaction mixture as solid in 33.3% yield; ^{1}H NMR (400 MHz, DMSO-d_6) δ 10.59 (s, 1H), 9.61 (s, 1H), 9.15 (s, 1H), 8.79 (d, *J* = 4.1 Hz, 1H), 8.61 (d, *J* = 1.6 Hz, 1H), 8.54 (d, *J* = 7.9 Hz, 1H), 8.19 (d, *J* = 8.8 Hz, 1H), 8.01 (td, *J* = 7.8, 1.7 Hz, 1H), 7.97 (dd, *J* = 8.8, 1.9 Hz, 1H), 7.55 (dd, *J* = 6.6, 4.8 Hz, 1H), 2.74 (s, 3H); ^{13}C NMR (101 MHz, DMSO-d_6) δ 174.0, 160.6, 160.5, 155.4. 151.5, 150.3, 150.1, 149.6,

144.5, 137.7, 128.9, 125.5, 124.4, 122.7, 121.0, 115.8, 112.3, 12,7; HRMS (ESI^+) calculated for $C_{18}H_{13}N_5O_2$ $[M+H]^+$: 332.1069, found 332.5899.

3. Evaluation of IC_{50} and Selected Kinase Profiling

We used Reaction Biology Corp. *Kinase HotSpot*^{SM} service (www.reactionbiology.com) for IC_{50} determination of all compounds and kinase profile. Assay protocol: In a final reaction volume of 25 μL, Peptide substrate, [EAIYAAPFAKKK], 5 μM, ATP 10 μM, FLT3(h) (5-10 mU) is incubated with 25 mM Tris pH 7.5, 0.02 mM EGTA, 0.66 mg/mL myelin basic protein, 10 mM Mg Acetate and [γ-33P-ATP] (specific activity approx. 500 cpm/pmol, concentration as required). The reaction is initiated by the addition of the Mg-ATP mix. After incubation for 40 minutes at room temperature, the reaction is stopped by the addition of 5 μL of a 3% phosphoric acid solution. 10 μL of the reaction is then spotted onto a P30 filtermat and washed three times for 5 minutes in 75 mM phosphoric acid and once in methanol prior to drying and scintillation counting.

4. Copies of ^{1}H and ^{13}C NMR spectrum of selected compounds

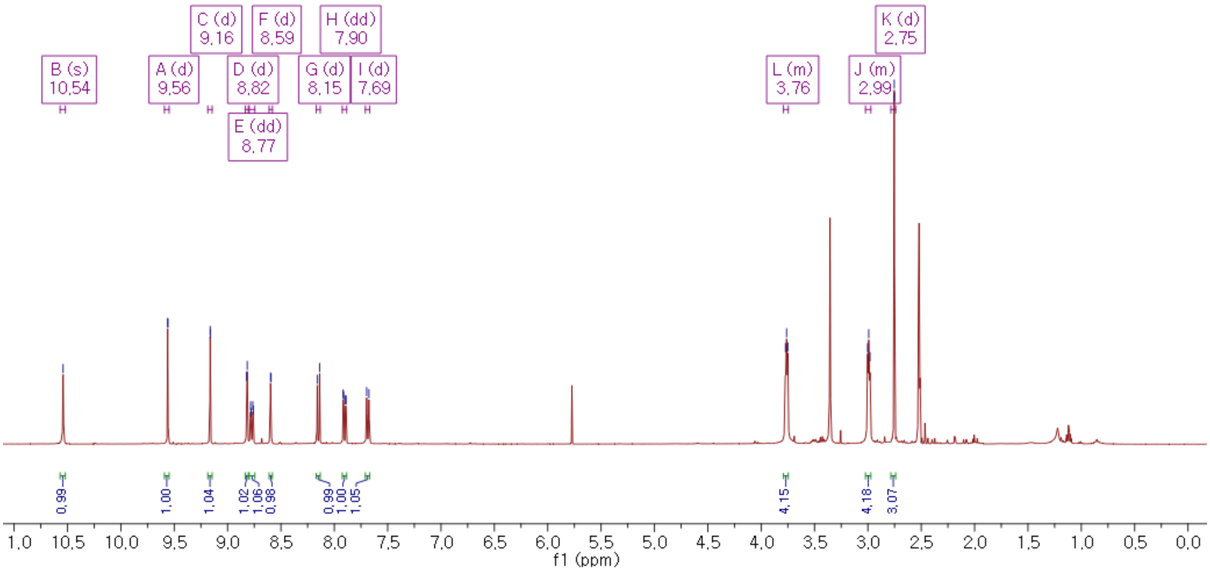

FigS1. ^{1}H NMR spectrum of compound **7a**

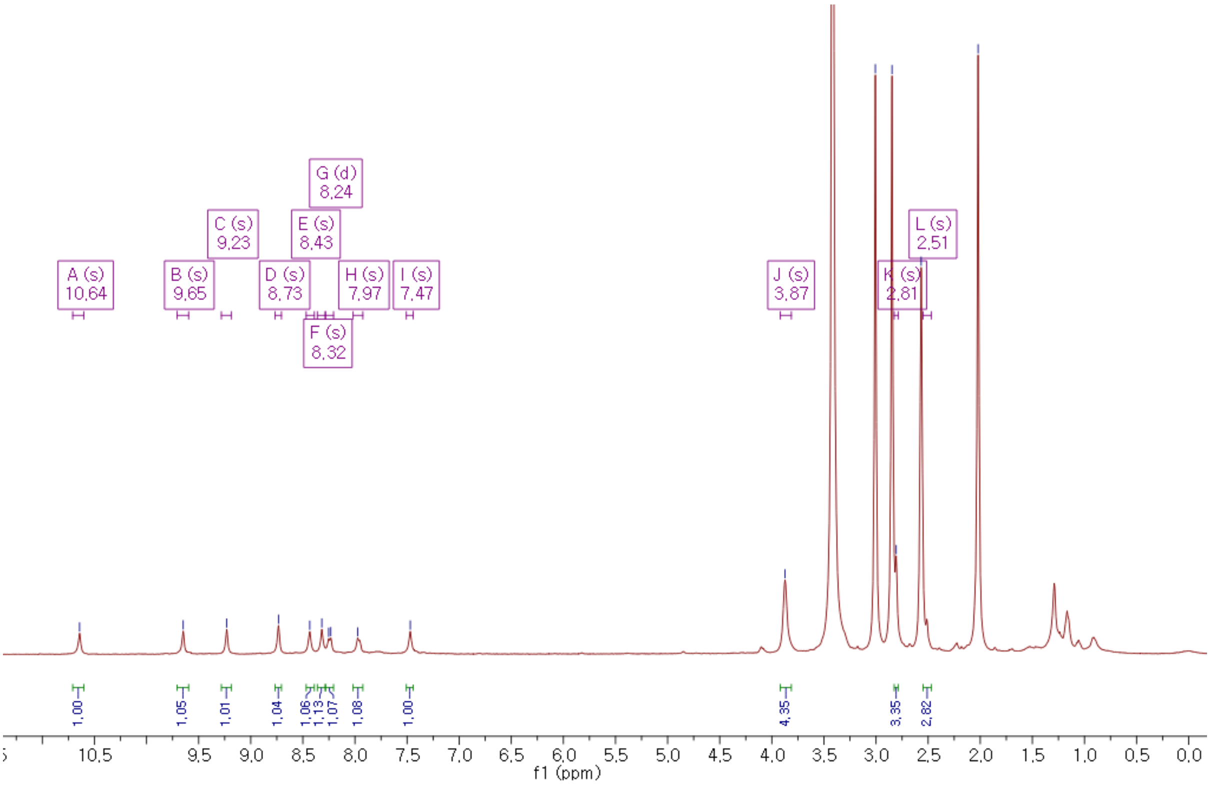

FigS2. ^{1}H NMR spectrum of compound **7b**

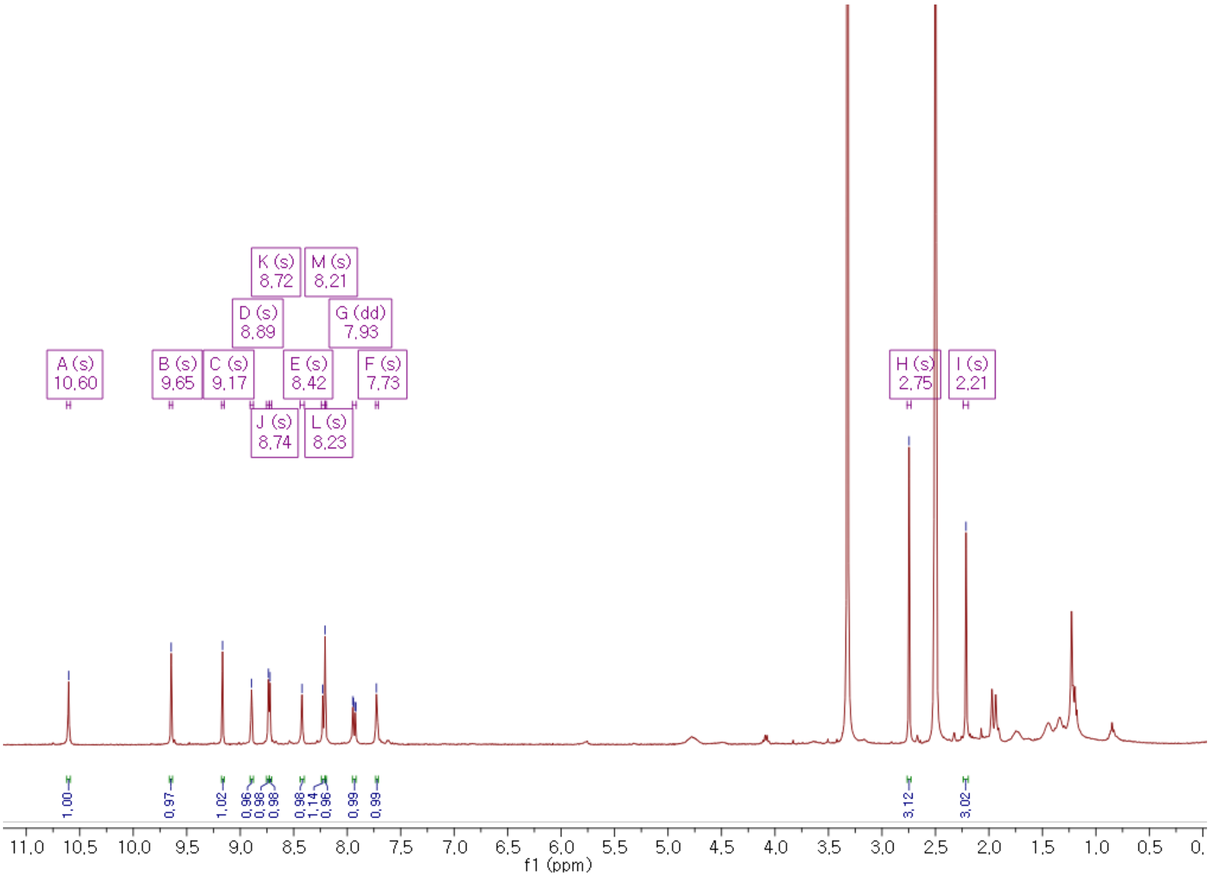

FigS3. ^{1}H NMR spectrum of compound **7c**

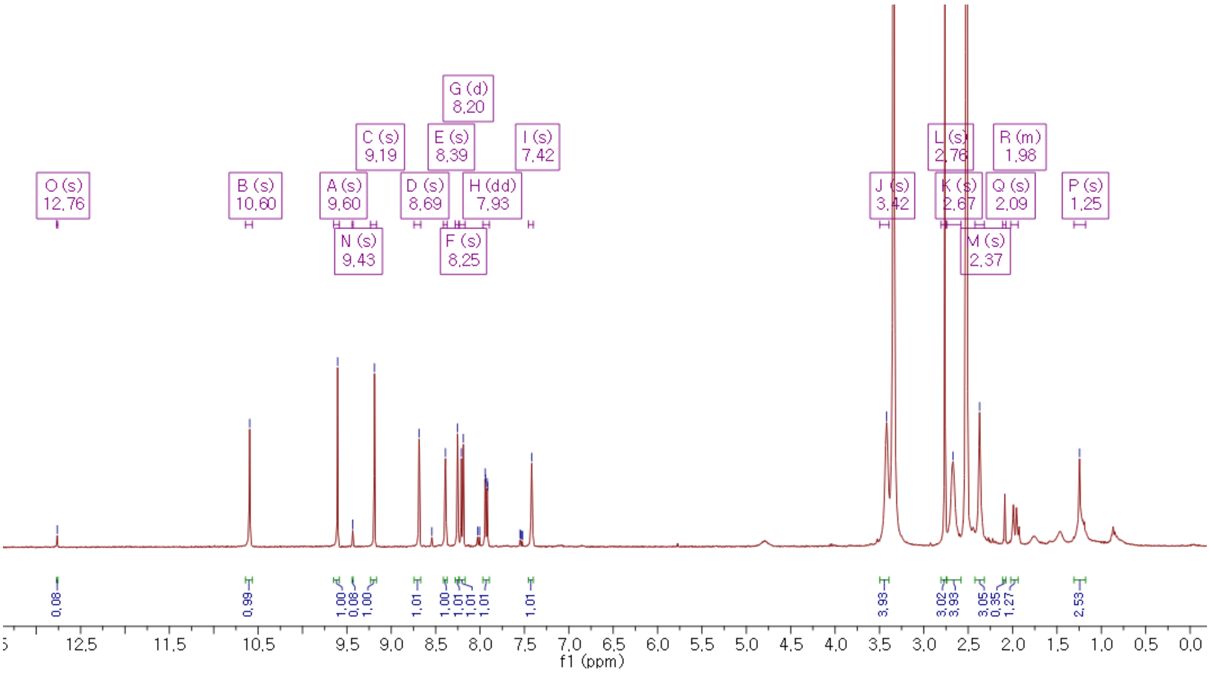

FigS4A. ^{1}H NMR spectrum of compound **7d**

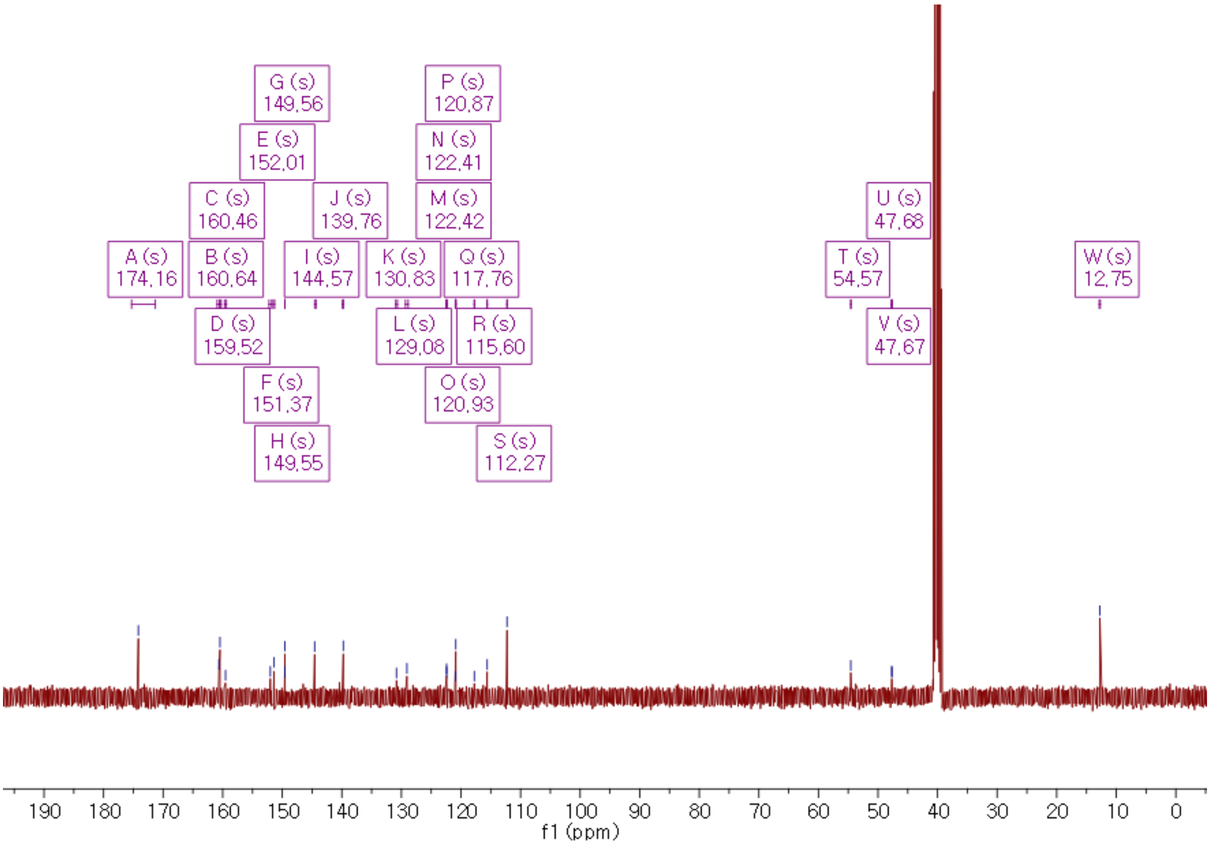

FigS4B. ^{13}C NMR spectrum of compound **7d**

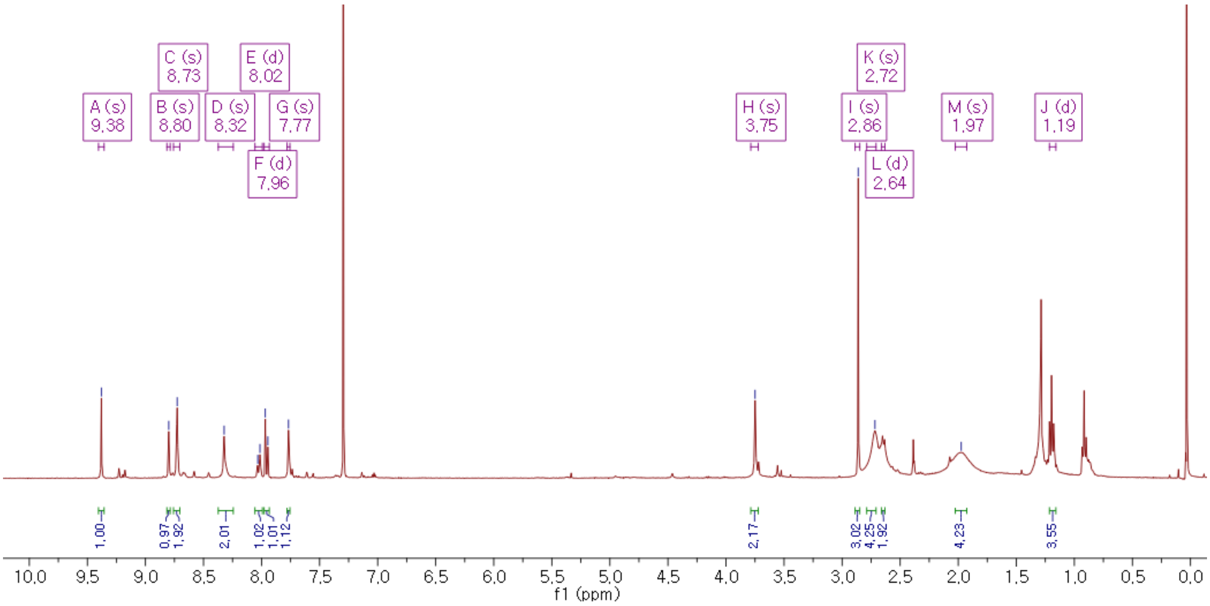

FigS5. ^{1}H NMR spectrum of compound **7e**

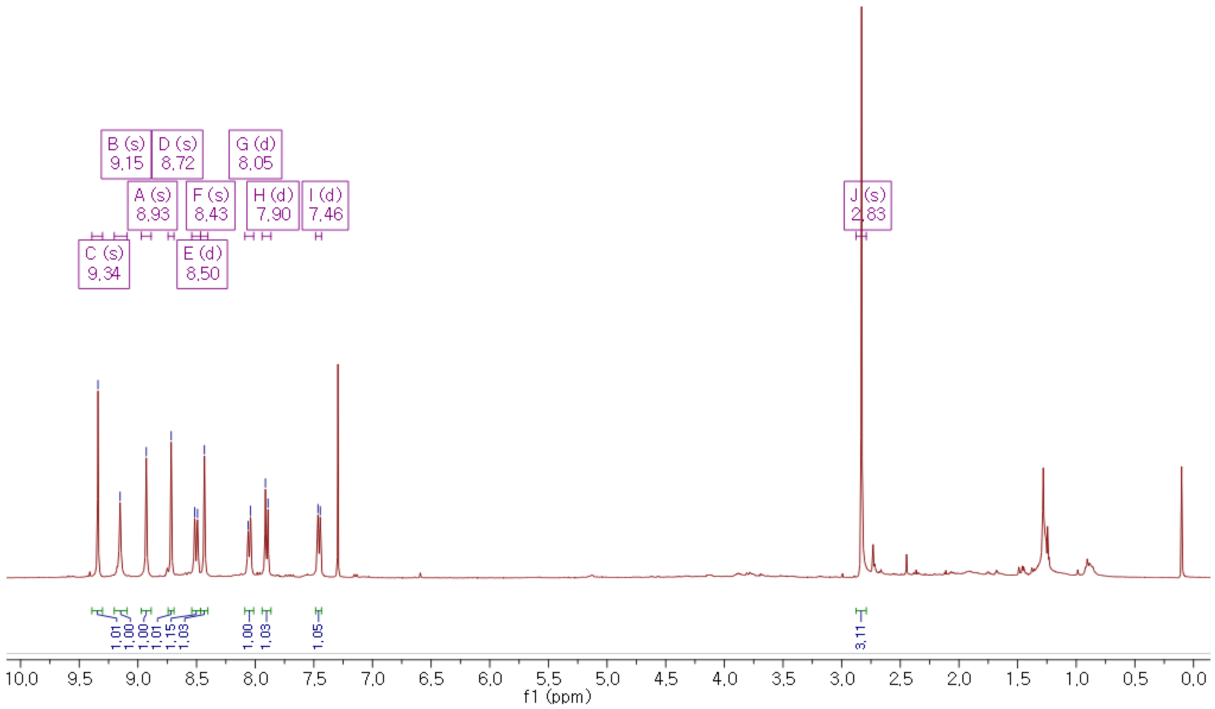

FigS6. ¹H NMR spectrum of compound **7f**

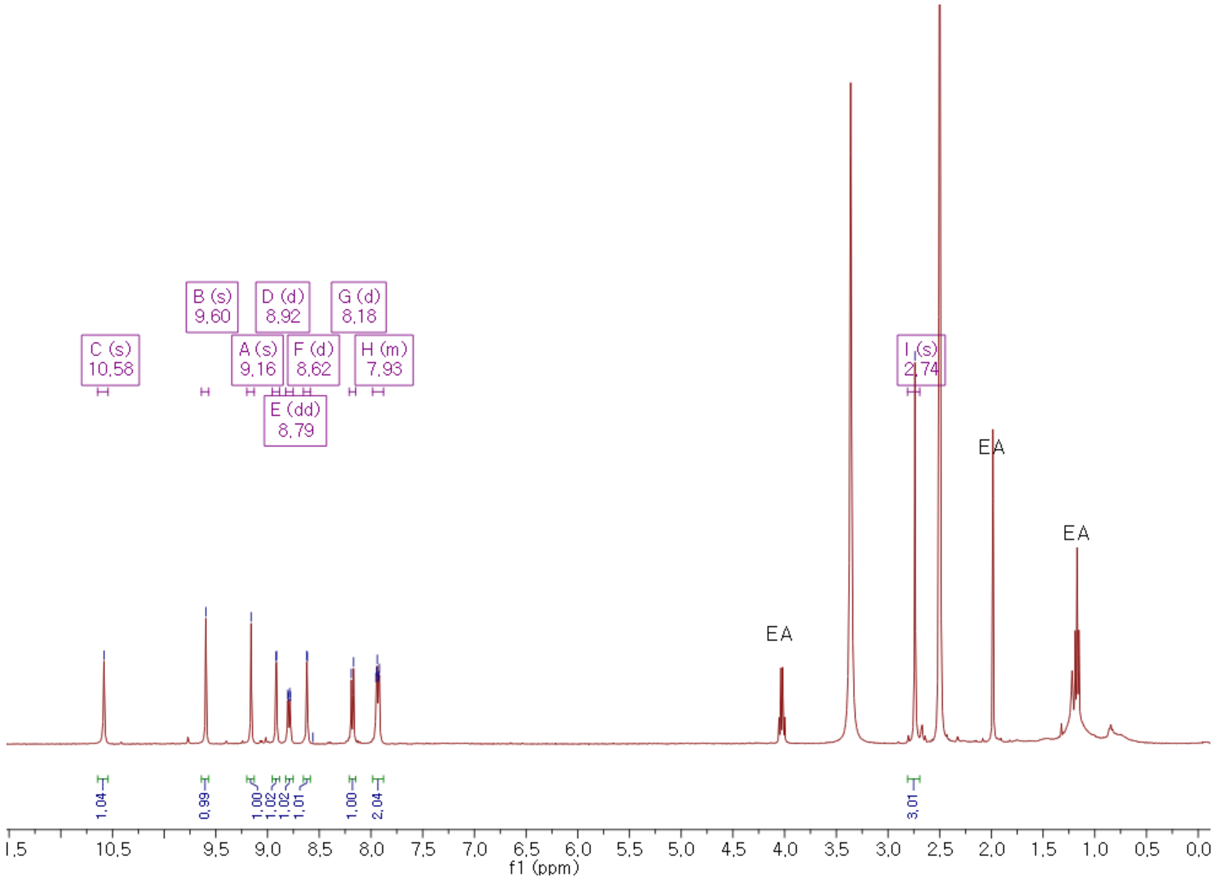

FigS7. ¹H NMR spectrum of compound **7g**

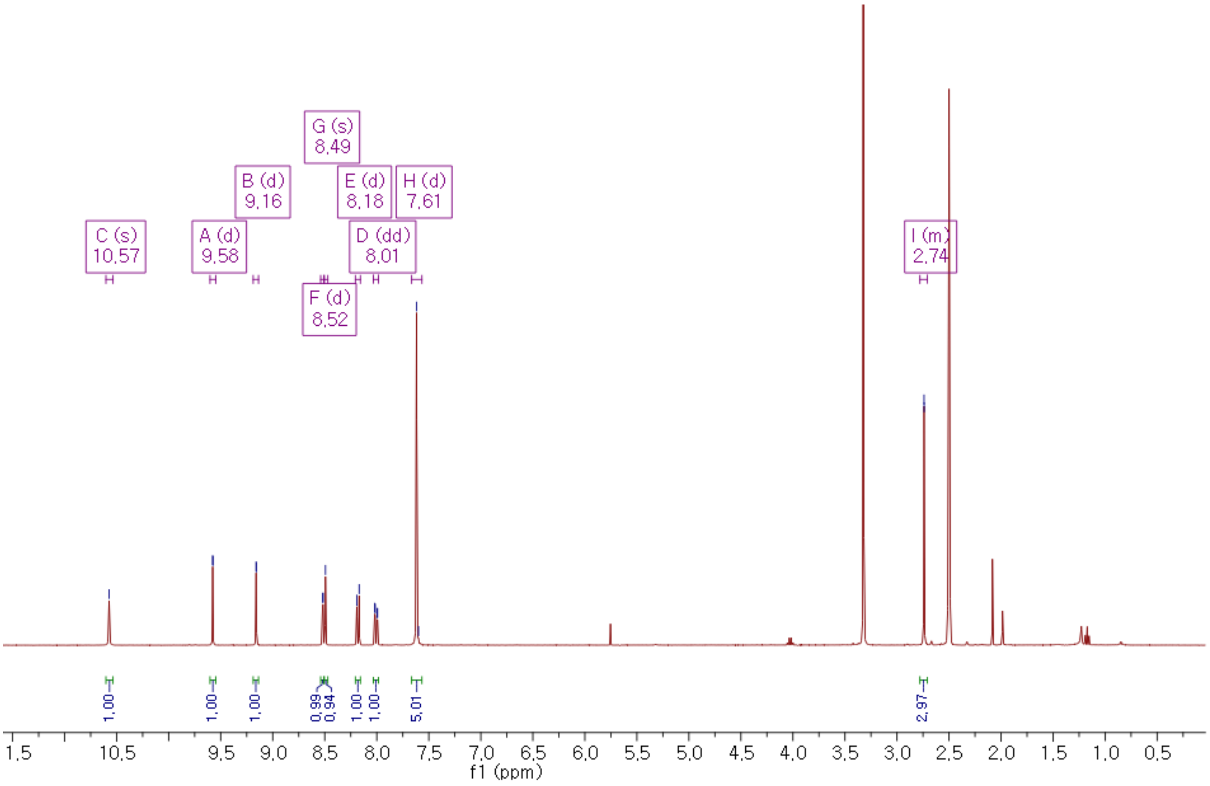

FigS8. ^{1}H NMR spectrum of compound **7h**

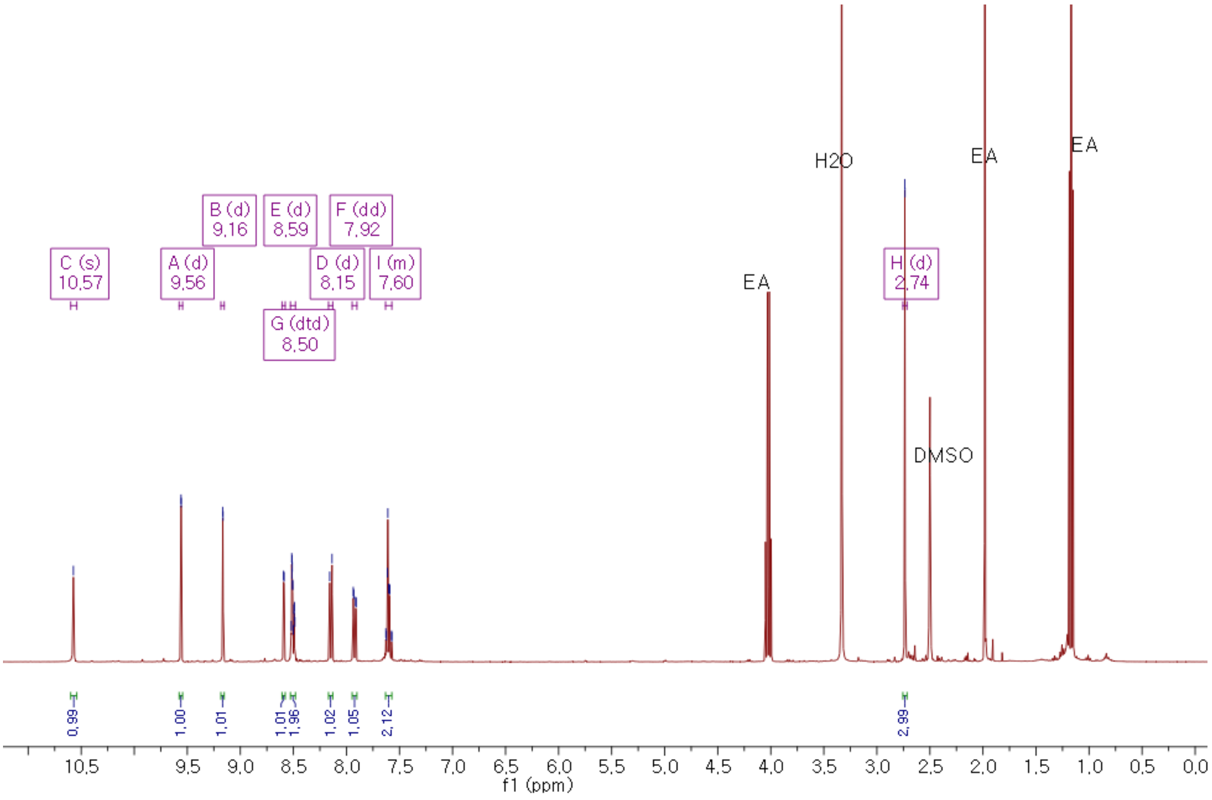

FigS10. ^{1}H NMR spectrum of compound **7i**

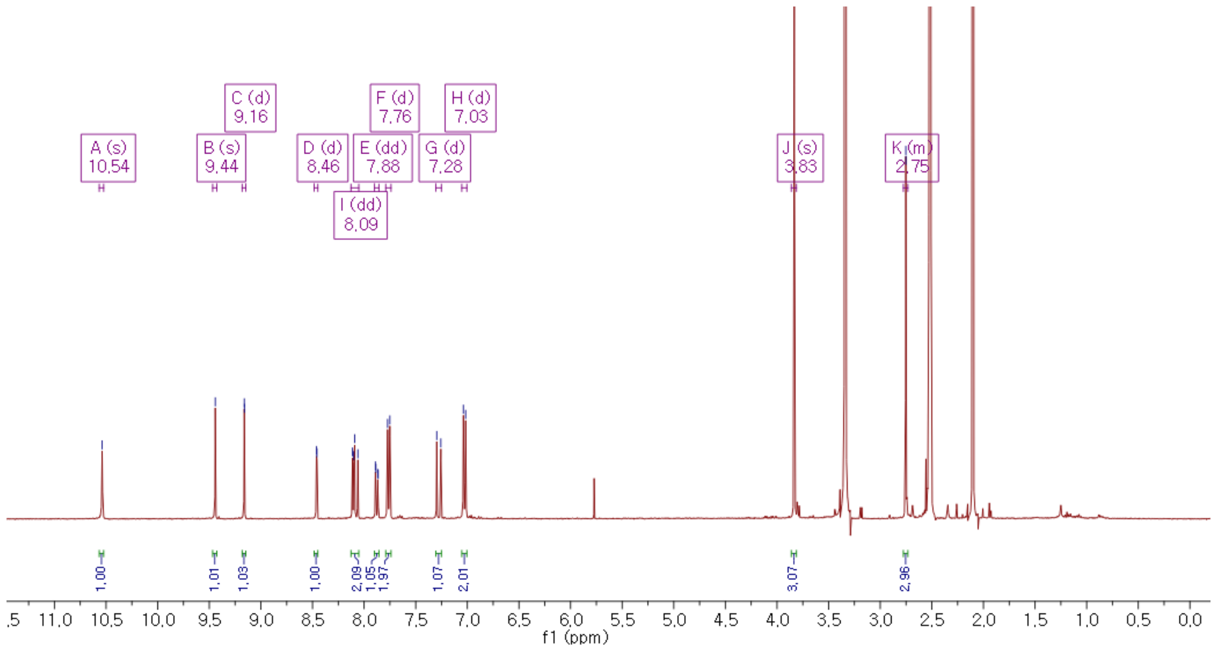

FigS11. ^{1}H NMR spectrum of compound **7j**

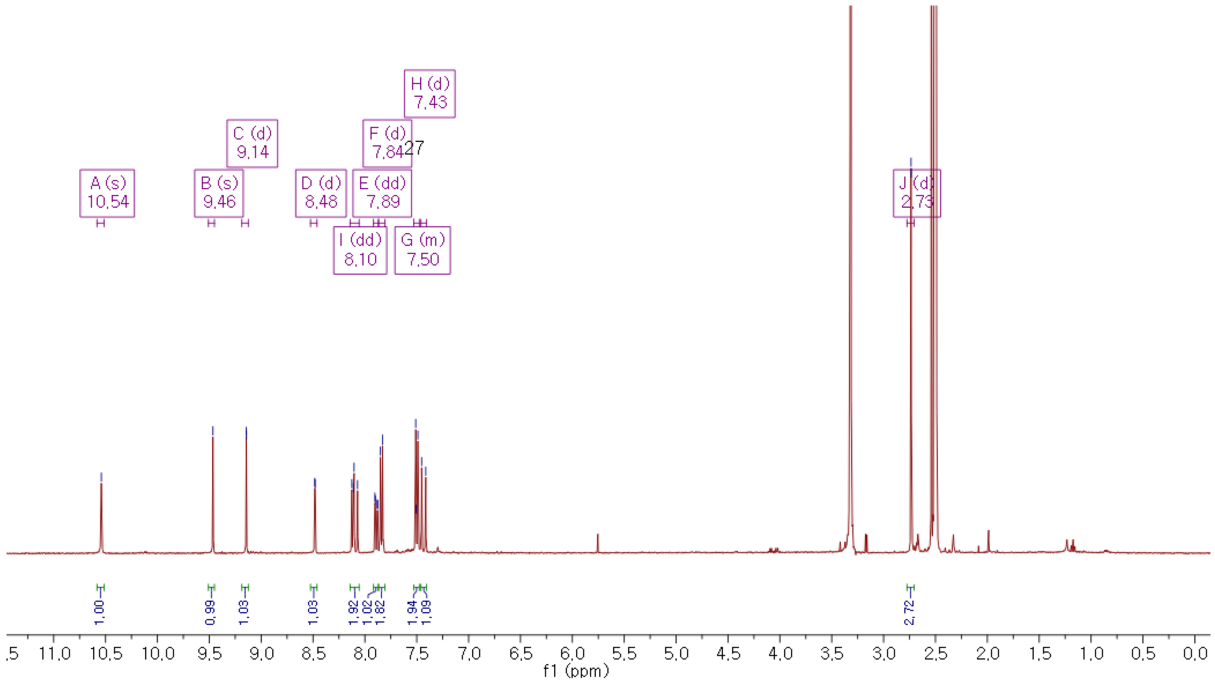

FigS12. ^{1}H NMR spectrum of compound **7k**

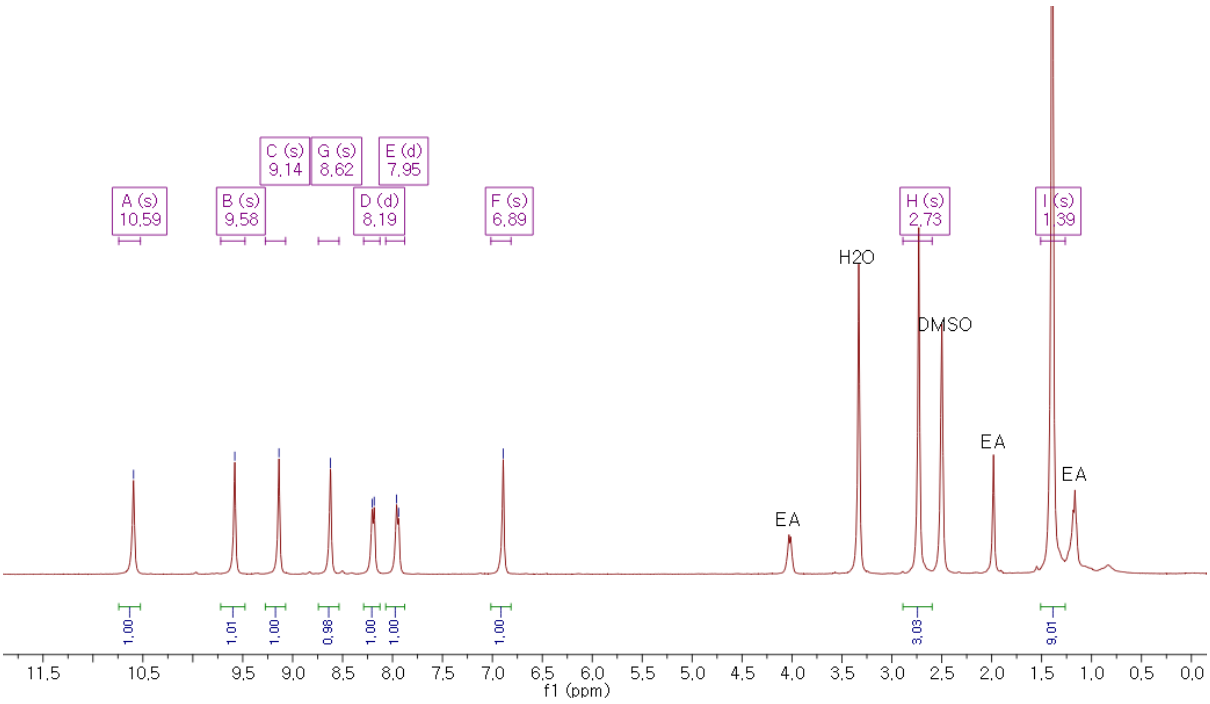

FigS13. ^{1}H NMR spectrum of compound **7l**

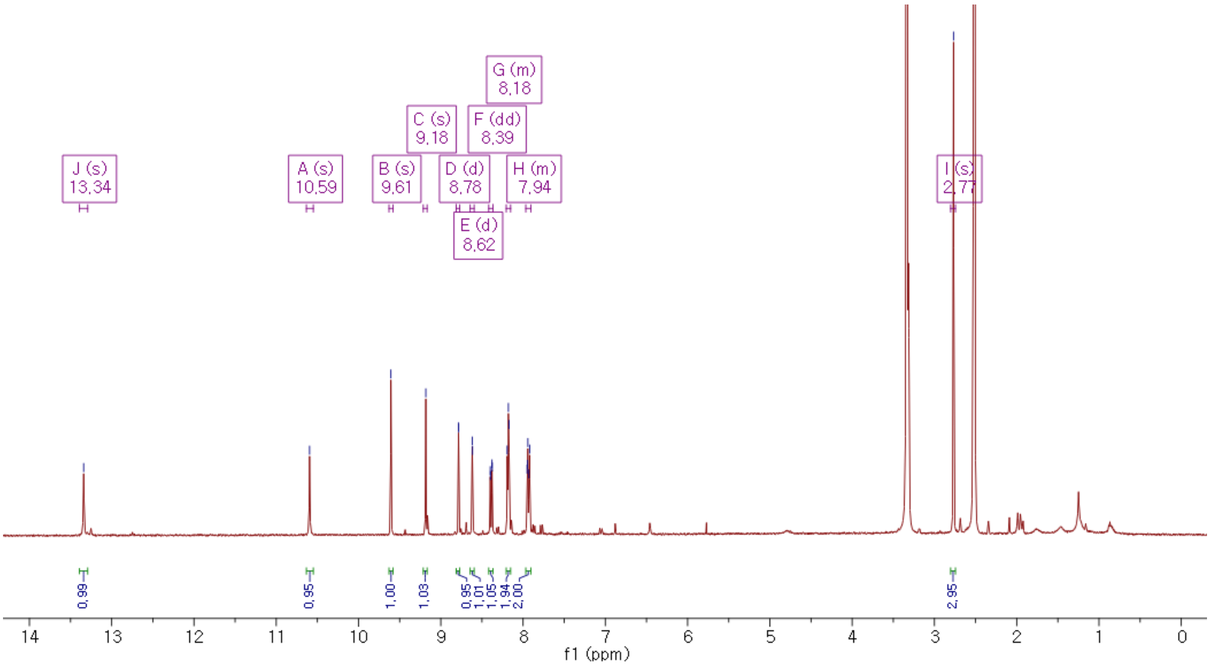

FigS14. ^{1}H NMR spectrum of compound **7m**

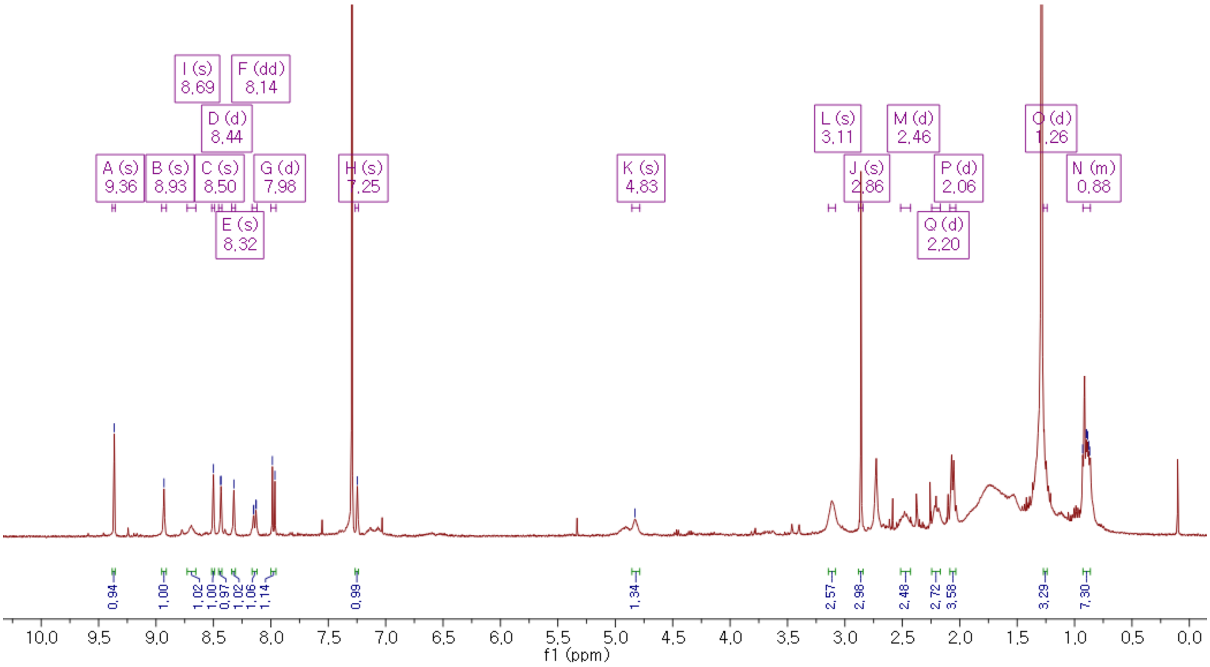

FigS15. ^{1}H NMR spectrum of compound **7n**

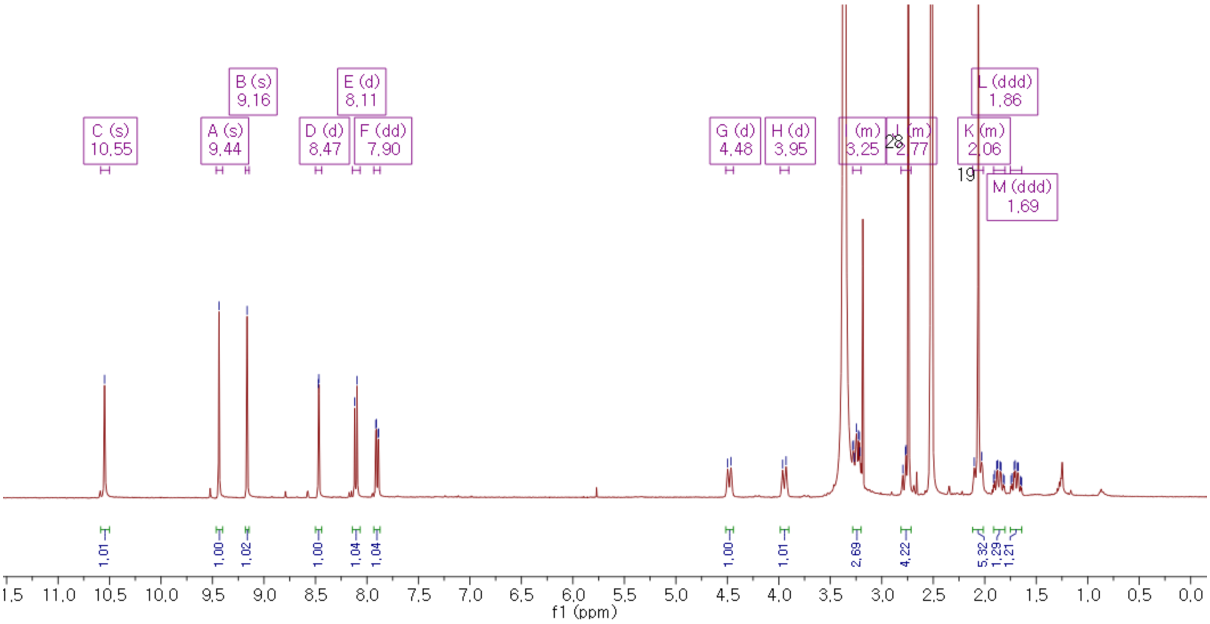

FigS16. ^{1}H NMR spectrum of compound **7o**

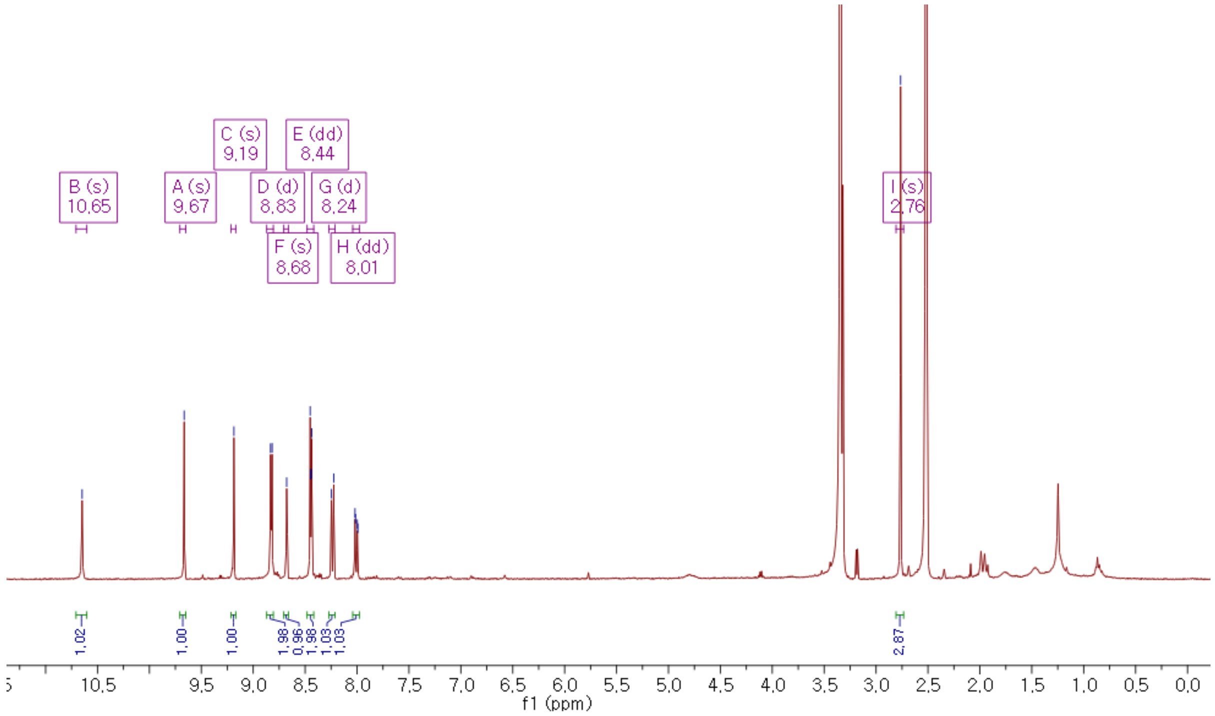

FigS17. ^{1}H NMR spectrum of compound **7p**

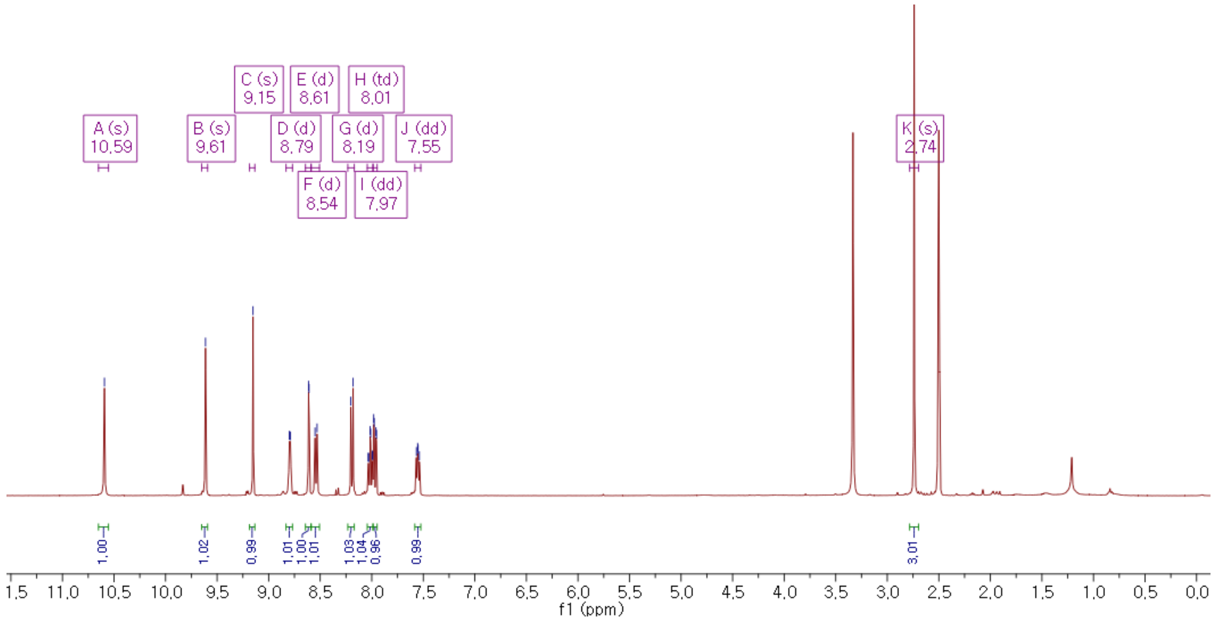

FigS18. ^{1}H NMR spectrum of compound **7q**

5. Percentages of enzymatic inhibition exerted by 7d and 7b toward selected protein kinases

Table S1. Percentages of enzymatic inhibition exerted by **7d** (10 μM) toward 36 selected protein kinases.

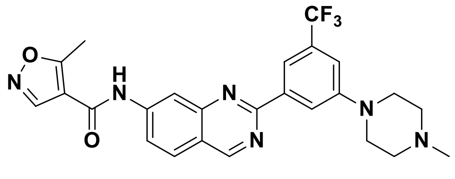

7d

Kinase	% Inhibition	Staurosporine IC_{50} (nM)
ABL1	6.46	31.0
AKT1	5.48	1.98
ALK	17.0	2.35
Aurora A	20.1	0.502
AXL	21.2	3.88
AXL (R499C)	10.1	3.21
BRAF (V599E)	5.19	6.79[a]
BTK	17.5	11.7
c-Kit	0	1.40
c-MET	13.2	57.8
c-Src	14.6	1.20
CAMKK1	0	59.6
CDK4/cyclin D1	3.01	30.4
EGFR	0	65.5
ERK1	15.0	4.47[b]
FGFR3	1.00	8.87
FLT1/VEGFR1	5.23	5.65
FLT3	96.6	1.13
FLT3-ITD	95.2	1.58
FMS	5.49	1.34
FYN	19.0	1.07
GSK3b	8.06	4.4
IGF1R	00	31.7
JAK3	0.80	0.0784
JNK3	19.5	65.8[c]

KDR/VEGFR2	16.8	11.4
LCK	19.7	1.39
LYN	20.5	0.675
MEK1	0	14.7
P38a/MAPK14	0	16.0[d]
PKA	2.5	1.37
PLK1	0	111
RIPK3	12.8	1650[a]
RON/MST1R	13.5	140
ROS/ROS1	14.4	0.174
SYK	22.4	0.436

[a] Data for GW5074[1]

[b] Data for SCH772984[2,3]

[c] Data for JNKI VIII[4, 5]

[d] Data for SB202190[6]

FigS19. Percentages of enzymatic inhibition exerted by **7b** toward 35 selected protein kinases

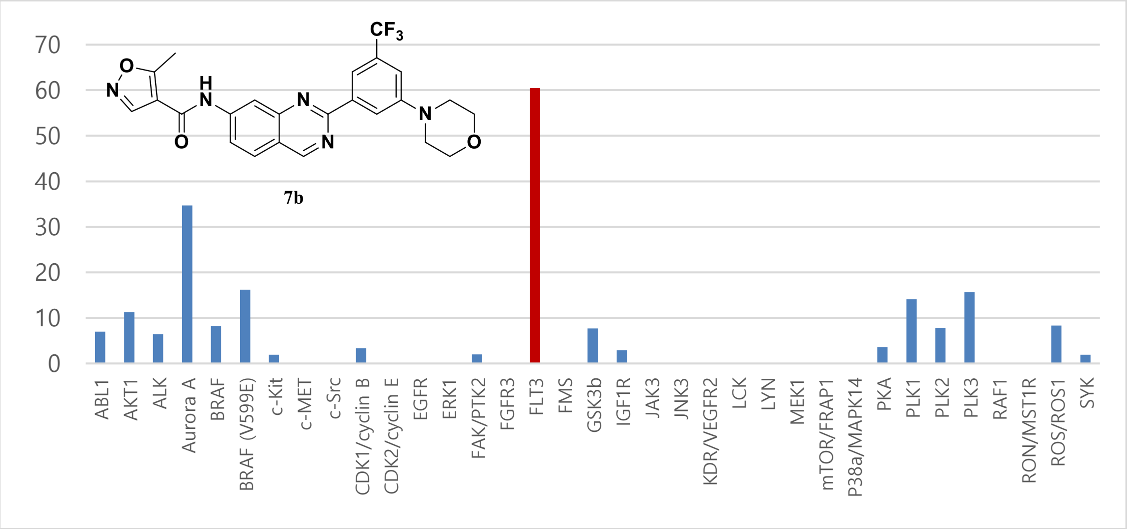

6. References

1. Lackey K, Cory M, Davis R, Frye S V., Harris PA, Hunter RN, et al. The discovery of potent cRaf1 kinase inhibitors. Bioorganic Med Chem Lett. 2000;10(3):223-226.
2. Seger R, Krebs EG. The MAPK signaling cascade. *FASEB. J.* 1995;9(9):726-735.
3. Morris EJ, Jha S, Restaino CR, Dayananth P, Zhu H, Cooper A, et al. Discovery of a novel ERK inhibitor with activity in models of acquired resistance to BRAF and MEK inhibitors. *Cancer Discov.* 2013;3(7):742–750.
4. Vivanco I, Palaskas N, Tran C, Finn SP, Getz G, Kennedy NJ, et al. Identification of the JNK Signaling Pathway as a Functional Target of the Tumor Suppressor PTEN. *Cancer Cell* 2007;11(6):555-569.
5. Szczepankiewicz BG, Kosogof C, Nelson LTJ, Liu G, Liu B, Zhao H, et al. Aminopyridine-based c-Jun N-terminal kinase inhibitors with cellular activity and minimal cross-kinase activity. *J. Med. Chem.* 2006;49(12):3563-3580.
6. Manthey CL, Wang SW, Kinney SD, Yao Z. SB202190, a selective inhibitor of p38 mitogen-activated protein kinase, is a powerful regulator of LPS-induced mRNAs in monocytes. *J. Leukoc. Biol.* 1998;64(3):409–417.
